# A Real‐World Pharmacovigilance Study of Fruquintinib Based on the FDA Adverse Event Reporting System (FAERS) Database

**DOI:** 10.1002/cam4.71352

**Published:** 2025-11-07

**Authors:** Yajing Xu, Dongchen Wang, Yuyan Xu

**Affiliations:** ^1^ Department of Radiation Oncology Wenzhou Central Hospital Wenzhou China; ^2^ Department of Otorhinolaryngology‐Head and Neck Surgery Shenzhen University General Hospital Shenzhen China; ^3^ Department of Radiotherapy Tongde Hospital of Zhejiang Province Hangzhou China

**Keywords:** adverse events, FAERS database, fruquintinib, oncology, pharmacovigilance

## Abstract

**Background:**

Fruquintinib is a highly selective small‐molecule inhibitor that targets vascular endothelial growth factor receptors and is approved for the treatment of metastatic colorectal cancer. Given its increasing clinical adoption, a comprehensive pharmacovigilance evaluation of the adverse events (AEs) is warranted.

**Methods:**

We conducted an analysis of fruquintinib‐related AEs by mining the FDA Adverse Event Reporting System (FAERS) database, covering the period from the fourth quarter of 2023 to the first quarter of 2025. The regulatory characterization of the AEs was based on the system organ class (SOC) and preferred term (PT) of the Medical Dictionary for Regulatory Activities (MedDRA). We employed four disproportionality analysis methods, including the reporting odds ratio (ROR), proportional reporting ratio (PRR), multi‐item gamma Poisson shrinker (MGPS), and Bayesian confidence propagation neural network (BCPNN). Furthermore, Weibull distribution modeling was employed to characterize the temporal risk patterns of adverse reactions.

**Results:**

Among 1632 reports in which fruquintinib was flagged as the primary suspect drug, 78 PTs met the criteria of all four algorithms. In addition to known AEs listed on the drug label, the analysis also identified previously unrecognized AEs, encompassing large intestinal obstruction, dehydration, peripheral neuropathy, renal‐limited thrombotic microangiopathy, and posterior reversible encephalopathy syndrome. The median onset time of fruquintinib‐associated AEs was 18 days, with 66.16% of cases occurring within the first month of treatment.

**Conclusion:**

This pharmacovigilance study aligned with established clinical observations. Additionally, novel AEs associated with fruquintinib therapy were detected, offering valuable evidence to enhance clinical surveillance strategies and risk assessment protocols.

## 1 Introduction

According to the latest 2025 global cancer statistics [1] and China's 2022 annual cancer registry report [2], colorectal cancer ranks among the top five malignancies in both incidence and mortality worldwide and nationally, posing a serious threat to public health and constituting a substantial disease burden. While overall colorectal cancer mortality continues to decline, emerging epidemiological trends reveal a concerning shift toward younger‐onset cases and more advanced‐stage diagnoses [3]. For patients with metastatic colorectal cancer (mCRC) refractory to standard first‐ and second‐line therapies, treatment options have historically been limited to third‐line trifluridine/tipiracil hydrochloride (TAS‐102) [4] and regorafenib [5], leaving those with progressive or intractable disease with few effective therapeutic alternatives.

Fruquintinib, an orally bioavailable small‐molecule tyrosine kinase inhibitor (TKI) with picomolar affinity for vascular endothelial growth factor (VEGF) receptors 1, 2, and 3, disrupts tumor angiogenesis and growth by blocking VEGF‐dependent phosphorylation and downstream signaling, thereby impairing endothelial cell proliferation, migration, and tube formation [6, 7]. This drug was first approved in China in September 2018 and demonstrated a significant survival benefit in the FRESCO‐2 trial [8], with successive approval by the U.S. Food and Drug Administration (FDA) in November 2023, the European Medicines Agency (EMA), and Japan in 2024 for the treatment of refractory mCRC. Available clinical data demonstrate that fruquintinib significantly improves both progression‐free survival (PFS) and overall survival (OS) in patients with refractory mCRC [8, 9]. Encouraging results have also been reported in clinical trials evaluating fruquintinib as monotherapy or in combination with other antineoplastic agents for treating various solid tumors, including endometrial carcinoma and gastric or gastroesophageal junction adenocarcinoma [10, 11]. However, despite these significant clinical benefits, the widespread clinical use of fruquintinib inevitably leads to treatment‐related adverse events (TRAEs) in patients.

Across the FRESCO phase III trial program of fruquintinib, the most prevalent but manageable TRAEs of fruquintinib included hypertension, palmar–plantar erythrodysesthesia syndrome (PPES), proteinuria, dysphonia, TSH level elevated, AST level elevated, bilirubin level elevated, diarrhea, ALT level elevated, stomatitis, decreased appetite, hypothyroidism, platelet count decreased, occult blood positive, fatigue, weight loss, asthenia, abdominal pain, nausea, constipation, vomiting, mucosal inflammation, arthralgia, and back pain [8, 9]. Dose reductions are most frequently triggered by PPES, proteinuria, or hypertension [12]. Clinically significant adverse reactions of special interest include severe hypertension, dermatological toxicity, proteinuria, gastrointestinal perforation, infection, hemorrhage, and thromboembolic events [8, 9, 13]. Additionally, isolated cases of fruquintinib‐associated posterior reversible encephalopathy syndrome (PRES) have been clinically reported [14, 15]. To address current evidence limitations in real‐world clinical settings, we implemented this pharmacovigilance study leveraging large‐scale postmarketing data and computational signal detection to evaluate the safety profile of fruquintinib.

The FDA Adverse Event Reporting System (FAERS) is a critical pharmacovigilance database maintained by the FDA to monitor postmarketing safety signals of approved drugs and therapeutic biologics [16, 17]. As a spontaneous reporting system, FAERS collects adverse event (AE) reports from healthcare professionals, consumers, and manufacturers, serving as a cornerstone for drug safety evaluation beyond clinical trials [18]. The utility of the FAERS database for postmarketing surveillance and adverse event signal detection is well established for common pharmaceuticals [19, 20]. This pharmacovigilance approach has been similarly successfully employed to characterize the safety profiles of diverse antineoplastic agents, spanning traditional chemotherapies to novel targeted and immunotherapeutic agents [21]—as evidenced by studies on agents such as vinca alkaloids [22], everolimus [23], and topotecan [24]. Grounded in this established methodological framework, our study seeks to evaluate the adverse event spectrum of fruquintinib using the FAERS database. Our study may improve real‐world drug safety surveillance of fruquintinib and facilitate evidence‐based prescribing practices among healthcare providers.

## 2 Materials and Methods

### 2.1 Data Source

A retrospective analysis of spontaneous AE reports was performed utilizing the open‐access FAERS repository (https://fis.fda.gov/extensions/FPD‐QDE‐FAERS/FPD‐QDE‐FAERS.html), focusing on fruquintinib‐related cases reported from Q4 2023 to Q1 2025. The generic name “fruquintinib” was used as the search term in DRUGNAME and PROD_AI. All data extraction, transformation, and analytical procedures were executed in the R programming environment (R version 4.4.3).

### 2.2 Data Processing and Standardization

**FIGURE 1:**
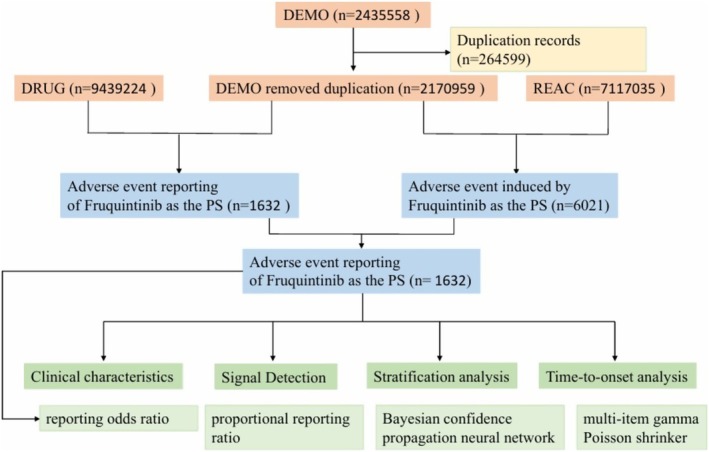
Pipeline for identifying fruquintinib‐related adverse events (AEs) from the FAERS database.

To address inherent duplicate reports in quarterly FAERS releases, we performed deduplication and prioritized the latest submissions. For reports with identical CASEIDs, only the record with the most recent FDA receipt date (FDA_DT) was retained. When CASEID and FDA_DT were identical, the entry with the highest PRIMARYID was preserved. Following deduplication, we retained only reports in which fruquintinib was designated the “primary suspect” drug for subsequent analysis. These reports were then extracted to construct our analytical dataset. All adverse drug events (ADEs) were subsequently standardized and categorized using preferred terms (PTs) and system organ classes (SOCs) as defined by the Medical Dictionary for Regulatory Activities (MedDRA). The multistep screening protocol of this study is illustrated in Figure 1.

### 2.3 Statistical Analysis

We employed disproportionality analysis to characterize potential associations between fruquintinib and AEs. Signal detection was performed using four quantitative pharmacovigilance algorithms: Two frequentist methods (reporting odds ratio [ROR] and proportional reporting ratio [PRR]), which demonstrate high sensitivity, and two Bayesian approaches (Bayesian confidence propagation neural network [BCPNN] and multi‐item gamma Poisson shrinker [MGPS]), which provide robust handling of sparse data [25, 26]. ROR significance required ≥ 3 cases with a 95% confidence interval (CI) lower bound > 1, while PRR significance mandated ≥ 3 cases, PRR ≥ 2, and chi‐square test value ≥ 4. The Bayesian frameworks provided additional validation, with BCPNN utilizing the information component's lower credibility bound (IC025 > 0) and the EBGM employing its fifth percentile threshold (EBGM05 > 2) as significance criteria. The multimethod approach enhances the robustness of results, with the signal strength directly proportional to the magnitude of each metric's output value (the calculation details are provided in Table 1).

**TABLE 1:** The specific formulas for the four algorithms used to evaluate the correlation between fruquintinib and AEs, Four major algorithms used for signal detection.

Algorithms	Equation	Criteria
ROR	ROR = ad/b/c	Lower limit of 95% CI > 1, *N* ≥ 3
	95% CI = e^ln(ROR) ± 1.96(1/a + 1/b + 1/c + 1/d)^0.5^	
PRR	PRR = a(c + d)/c/(a + b)	PRR ≥ 2, χ^2^ ≥ 4, *N* ≥ 3
	χ^2^ = [(ad‐bc)^2](a + b + c + d)/[(a + b)(c + d)(a + c)(b + d)]	
BCPNN	IC = log_2_a(a + b + c + d)(a + c)(a + b)	IC025 > 0
	95% CI = E(IC) ± 2 V(IC)^0.5	
MGPS	EBGM = a(a + b + c + d)/(a + c)/(a + b)	EBGM05 > 2
	95% CI = e^ln(EBGM) ± 1.96(1/a + 1/b + 1/c + 1/d)^0.5^	

*Note:* Equation: a, number of reports containing both the target drug and target adverse drug reaction; b, number of reports containing other adverse drug reaction of the target drug; c, number of reports containing the target adverse drug reaction of other drugs; d, number of reports containing other drugs and other adverse drug reactions. 95% CI, 95% confidence interval; N, the number of reports; χ^2^, chi‐squared; IC, information component; IC025, the lower limit of 95% CI of the IC; E(IC), the IC expectations; V(IC), the variance of IC; EBGM, empirical Bayesian geometric mean; EBGM05, the lower limit of 95% CI of EBGM.

The latency period for AEs was calculated as the temporal interval between drug initiation (START_DT) and adverse event manifestation (EVENT_DT). To ensure data quality, reports with chronologically implausible dates (EVENT_DT preceding START_DT) or inconsistent temporal documentation were systematically excluded from analysis. The time‐to‐event data for adverse reactions were subjected to Weibull survival analysis, allowing characterization of both early‐ and delayed‐onset patterns.

## 3 Results

### 3.1 Descriptive Analysis

Our study included 2,170,959 eligible reports submitted to FAERS between Q4 2023 and Q1 2025. Fruquintinib was designated as a PS agent in 1632 documented cases. Table 2 summarizes the comprehensive safety profile of fruquintinib‐associated AEs. Key demographic characteristics revealed a male predominance (51.9% vs. 41.1% female), with the majority of cases occurring in adults aged 18–65 years (24.4%), while patients aged 65–85 years accounted for 19.6% of the sample. In terms of weight composition, the majority of patients weighed between 50 and 100 kg. Death (33.9%) and other serious medical events (39.4%) represented the most frequent adverse outcomes, while death may reflect underlying disease progression rather than direct drug toxicity. Other severe outcomes included hospitalization (24.8%), life‐threatening (1.3%) and disability (0.6%). Geographically, the majority of reports originated from the United States (62.9%), with China (14.6%), Japan (8.1%), Great Britain (3%), and France (2.5%) comprising smaller proportions. Notably, 34% of the reports were submitted by consumers, highlighting substantial patient engagement in pharmacovigilance. Following fruquintinib, which was approved by the FDA in 2023, temporal analysis demonstrated a progressive increase in AE reports, reaching 67.2% of the total documented cases by 2024. Preliminary 2025 data (Q1) suggested that this upward trajectory may persist, although complete annual reporting remains pending.

**TABLE 2:** Clinical characteristics of reports with fruquintinib from the FAERS database (Q4 2023 to Q1 2025).

Characteristics	Case number, *n*	Case proportion, %
Gender		
Female	671	41.1
Male	847	51.9
Unknown	114	7
Age		
< 18	92	5.6
18 ~ 64.9	399	24.4
65 ~ 85	320	19.6
> 85	12	0.7
Missing	809	49.6
Weight		
< 50 kg	45	2.8
50 ~ 100 kg	239	14.6
> 100 kg	25	1.5
Missing	1323	81.1
Outcome		
Death	554	33.9
Hospitalization	405	24.8
Life‐Threatening	21	1.3
Disability	9	0.6
Other	643	39.4
Reported Countries (Top five)		
US	1027	62.9
CN	238	14.6
JP	132	8.1
GB	49	3
FR	41	2.5
Reporter type		
Consumer	555	34
Physician	360	22.1
Health Professional	208	12.7
Pharmacist	105	6.4
Missing	404	24.8
Reporting year		
2023	39	2.4
2024	1097	67.2
2025	496	30.4

### 3.2 Signals of System Organ Class

**FIGURE 2:**
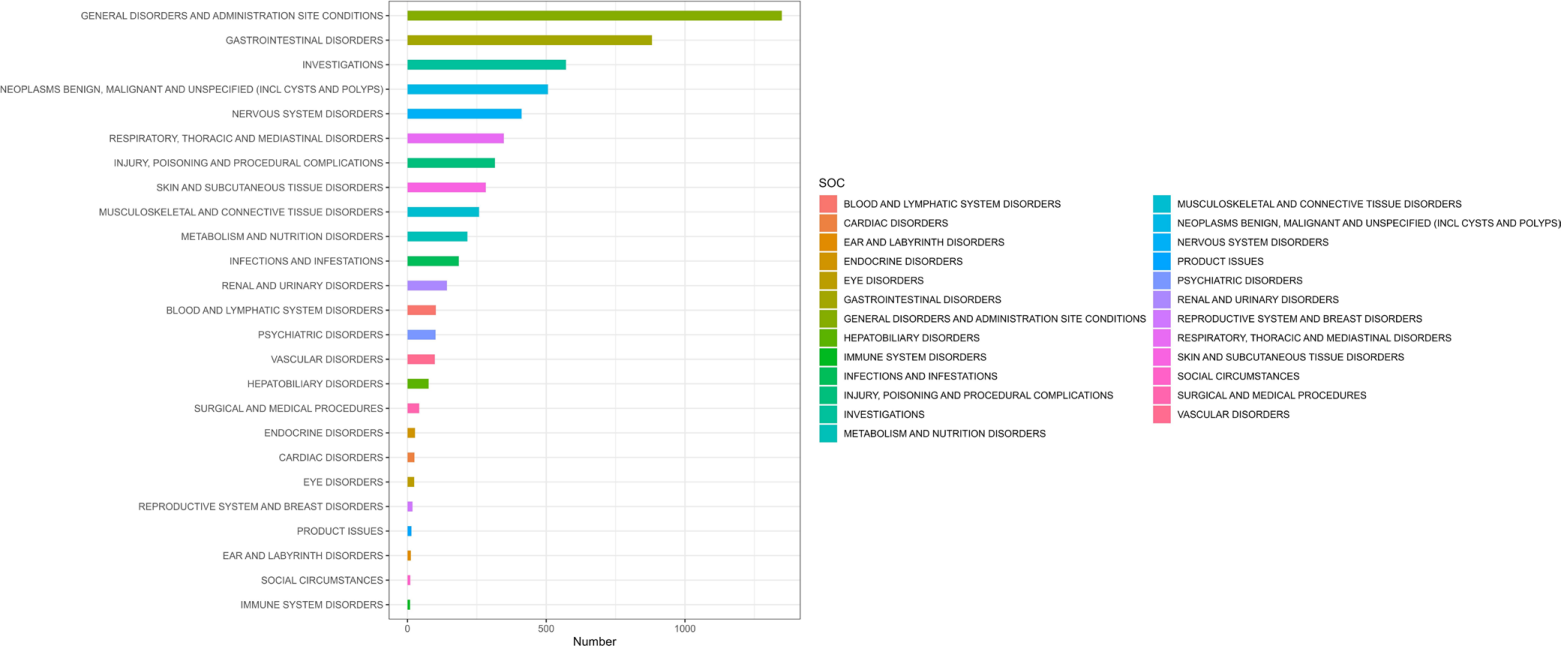
AEs related to the system organ class (SOC) level.

**FIGURE 3:**
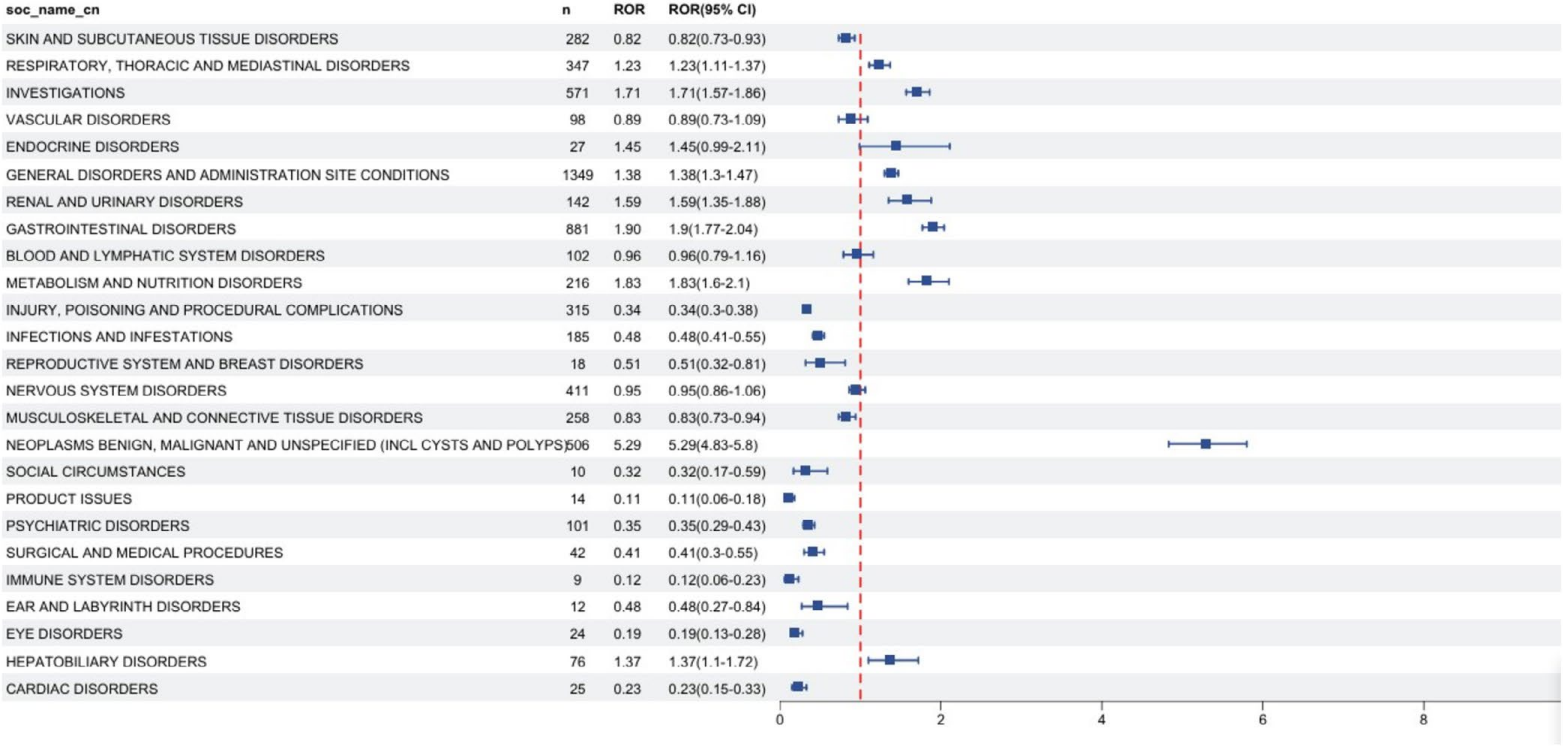
Forest plot presenting the reporting odds ratio (ROR) of AE‐related SOCs.

Table 3 details the signal intensities of fruquintinib‐associated AEs spanning 25 SOCs, only “Neoplasms benign, malignant and unspecified (incl cysts and polyps)” (*n* = 506; ROR = 5.29, PRR = 4.93, EBGM = 4.91, IC = 2.3) satisfied all four predefined criteria. Notably, among the significant SOCs identified, respiratory/thoracic/mediastinal disorders, investigations, general disorders/administration site conditions, renal/urinary disorders, gastrointestinal disorders, metabolism/nutrition disorders, and hepatobiliary disorders were consistently highlighted by at least two of the four evaluation indices. Figure 2 presents the descending frequency distribution of AEs by SOC, with the highest incidence observed in “General disorders and administration site conditions” (*n* = 506; ROR = 5.29, PRR = 4.93, EBGM = 4.91, IC = 2.30). Figure 3 illustrates the signal strengths of fruquintinib‐associated AEs across SOCs, as quantified using the ROR method, the most sensitive of our disproportionality analyses. Signal robustness showed a positive correlation with the magnitude of ROR.

**TABLE 3:** Signal values of reports associated with fruquintinib at the SOC level.

System organ class (soc)	Cases	ROR (95% two‐sided ci)	PRR (χ^2^)	EBGM (EBGM05)	IC (IC025)
Skin and subcutaneous tissue disorders	282	0.82 (0.73–0.93)	0.83 (10.13)	0.83 (0.75)	−0.27 (−0.44)
Respiratory, thoracic and mediastinal disorders	347	1.23 (1.11–1.37)	1.22 (14.34)	1.22 (1.11)	0.29 (0.13)
Investigations	571	1.71 (1.57–1.86)	1.64 (151.39)	1.64 (1.53)	0.71 (0.59)
Vascular disorders	98	0.89 (0.73–1.09)	0.9 (1.21)	0.9 (0.76)	−0.16 (−0.45)
Endocrine disorders	27	1.45 (0.99–2.11)	1.44 (3.68)	1.44 (1.05)	0.53 (−0.02)
General disorders and administration site conditions	1349	1.38 (1.3–1.47)	1.3 (110.83)	1.3 (1.23)	0.37 (0.29)
Renal and urinary disorders	142	1.59 (1.35–1.88)	1.58 (30.71)	1.58 (1.37)	0.66 (0.42)
Gastrointestinal disorders	881	1.9 (1.77–2.04)	1.77 (319.65)	1.77 (1.66)	0.82 (0.72)
Blood and lymphatic system disorders	102	0.96 (0.79–1.16)	0.96 (0.2)	0.96 (0.81)	−0.06 (−0.35)
Metabolism and nutrition disorders	216	1.83 (1.6–2.1)	1.8 (78.55)	1.8 (1.61)	0.85 (0.65)
Injury, poisoning, and procedural complications	315	0.34 (0.3–0.38)	0.37 (393.51)	0.37 (0.34)	−1.43 (−1.6)
Infections and infestations	185	0.48 (0.41–0.55)	0.49 (102.87)	0.49 (0.44)	−1.02 (−1.23)
Reproductive system and breast disorders	18	0.51 (0.32–0.81)	0.51 (8.53)	0.51 (0.35)	−0.97 (−1.63)
Nervous system disorders	411	0.95 (0.86–1.06)	0.96 (0.82)	0.96 (0.88)	−0.06 (−0.21)
Musculoskeletal and connective tissue disorders	258	0.83 (0.73–0.94)	0.84 (8.76)	0.84 (0.75)	−0.26 (−0.44)
Neoplasms, benign, malignant, and unspecified (including cysts and polyps)	506	5.29 (4.83–5.8)	4.93 (1606.7)	4.91 (4.55)	2.3 (2.16)
Social circumstances	10	0.32 (0.17–0.59)	0.32 (14.54)	0.32 (0.19)	−1.64 (−2.51)
Product issues	14	0.11 (0.06–0.18)	0.11 (106.44)	0.11 (0.07)	−3.22 (−3.97)
Psychiatric disorders	101	0.35 (0.29–0.43)	0.36 (117.25)	0.36 (0.31)	−1.46 (−1.74)
Surgical and medical procedures	42	0.41 (0.3–0.55)	0.41 (36.34)	0.41 (0.32)	−1.29 (−1.73)
Immune system disorders	9	0.12 (0.06–0.23)	0.12 (57.15)	0.12 (0.07)	−3.03 (−3.94)
Ear and labyrinth disorders	12	0.48 (0.27–0.8)	0.48 (6.9)	0.48 (0.3)	−1.07 (−1.87)
Eye disorders	24	0.19 (0.13–0.28)	0.19 (83.17)	0.19 (0.14)	−2.38 (−2.96)
Hepatobiliary disorders	76	1.37 (1.1–1.72)	1.37 (7.66)	1.37 (1.13)	0.45 (0.12)
Cardiac disorders	25	0.23 (0.15–0.33)	0.23 (66.08)	0.23 (0.16)	−2.13 (−2.69)

### 3.3 Signals of Preferred Terms

**FIGURE 4:**
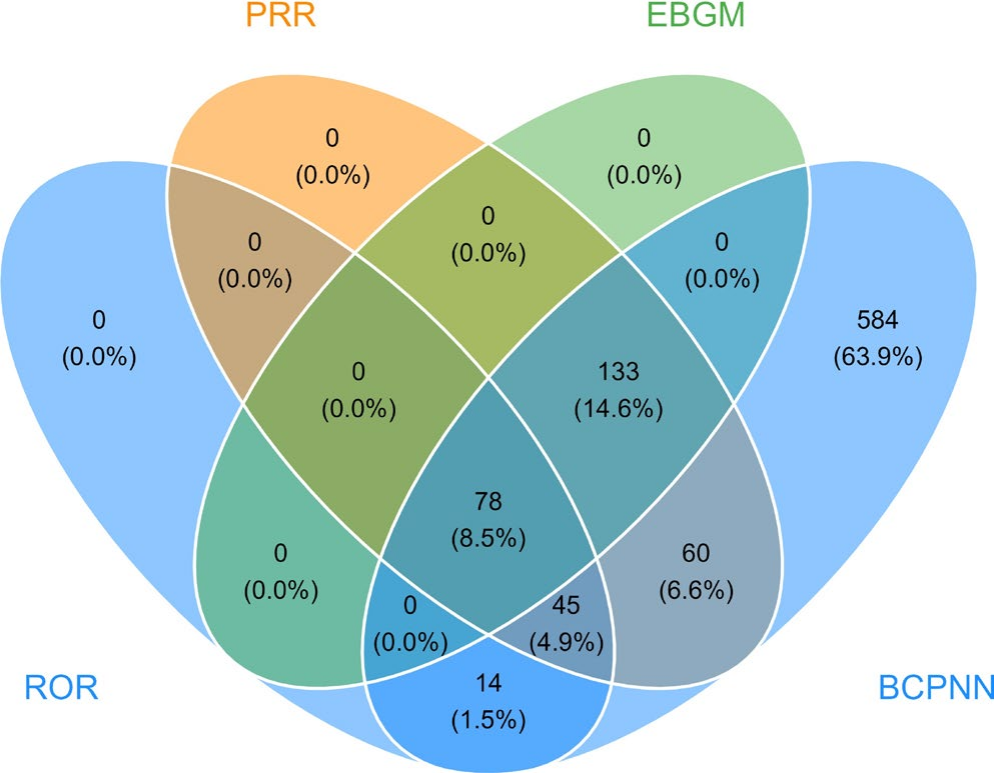
Venn diagram for screening significant PTs according to the four algorithms.

We employed four distinct signal detection algorithms to identify potential fruquintinib‐related AEs. A total of 137 PTs were detected by the ROR method, along with 316 PTs determined via the PRR method, 211 PTs quantified by the EBGB method, and 914 PTs derived from the BCPNN method. After consolidation, 78 significant PTs were validated (Figure 4).

Following the exclusion of neoplasms, benign, malignant, and unspecified (including cysts and polyps) that could reflect disease progression rather than drug effects, we identified 60 statistically significant PTs meeting all four algorithm criteria (Table 4). Excluding death cases (*n* = 438) potentially attributable to tumor progression, the AE profile revealed expected patterns: fatigue (*n* = 248), blood pressure increased (*n* = 173), asthenia (*n* = 130), decreased appetite (*n* = 127), dysphonia (*n* = 123), myelosuppression (*n* = 77), stomatitis (*n* = 56), hypertension (*n* = 53), abdominal pain upper (*n* = 52), and PPES (*n* = 51), which were aligned with label information and previous trial data. Our analysis revealed unexpected significant AEs, including peripheral neuropathy, dehydration, ascites, hypersomnia, chromaturia, biliary obstruction, anal abscess, increased ammonia, decreased urine output, large intestinal obstruction, renal‐limited thrombotic microangiopathy (TMA), PRES, and so on.

**TABLE 4:** Signal strength of reports of Fruquintinib at the Preferred Term (PT) level in FAERS database.

SOC	Preferred terms (PTs)	Cases	ROR (95% CI)	PRR (χ_2_)	EBGM (EBGM05)	IC (IC025)
Blood and lymphatic system disorders	Myelosuppression	77	8.84 (7.06–11.08)	8.74 (524.75)	8.68 (7.19)	3.12 (2.79)
Endocrine disorders	Hypothyroidism	19	5.19 (3.31–8.15)	5.18 (63.83)	5.16 (3.54)	2.37 (1.72)
Gastrointestinal disorders	Stomatitis	56	8.32 (6.39–10.83)	8.25 (354.81)	8.2 (6.58)	3.04 (2.65)
	Abdominal pain upper	52	2.83 (2.15–3.72)	2.81 (60.84)	2.81 (2.24)	1.49 (1.09)
	Oral pain	29	15.78 (10.93–22.79)	15.71 (394.34)	15.52 (11.41)	3.96 (3.42)
	Ascites	16	6.57 (4.02–10.75)	6.56 (74.96)	6.53 (4.32)	2.71 (2)
	Rectal hemorrhage	13	3.8 (2.2–6.55)	3.79 (26.62)	3.78 (2.4)	1.92 (1.15)
	Gingival pain	7	10.41 (4.94–21.92)	10.4 (58.95)	10.32 (5.53)	3.37 (2.34)
	Tongue ulceration	7	28.25 (13.34–59.8)	28.22 (179.48)	27.58 (14.72)	4.79 (3.75)
	Oral discomfort	5	4.5 (1.87–10.84)	4.5 (13.57)	4.49 (2.15)	2.17 (0.98)
	Proctalgia	5	8.2 (3.4–19.76)	8.19 (31.35)	8.14 (3.9)	3.03 (1.84)
	Tongue discomfort	4	8.01 (3–21.42)	8.01 (24.36)	7.96 (3.5)	2.99 (1.7)
	Anal hemorrhage	3	12.27 (3.93–38.26)	12.26 (30.71)	12.14 (4.69)	3.6 (2.15)
	Intra‐abdominal fluid collection	3	13.85 (4.44–43.23)	13.84 (35.32)	13.69 (5.28)	3.78 (2.32)
	Gastrointestinal perforation	3	8.56 (2.75–26.66)	8.56 (19.88)	8.5 (3.29)	3.09 (1.64)
	Large intestinal obstruction	3	13.43 (4.3–41.91)	13.42 (34.1)	13.28 (5.12)	3.73 (2.28)
	Gastrointestinal motility disorder	3	5.28 (1.7–16.43)	5.28 (10.36)	5.26 (2.04)	2.4 (0.95)
General disorders and administration site conditions	Death	438	6.36 (5.77–7.02)	5.97 (1826.93)	5.95 (5.48)	2.57 (2.43)
	Fatigue	248	3.24 (2.85–3.68)	3.15 (367.67)	3.14 (2.83)	1.65 (1.47)
	Asthenia	130	3.86 (3.25–4.6)	3.8 (269.25)	3.79 (3.28)	1.92 (1.67)
	Mucosal inflammation	11	4.68 (2.59–8.46)	4.67 (31.63)	4.66 (2.84)	2.22 (1.38)
	Terminal state	4	7.37 (2.76–19.71)	7.37 (21.89)	7.33 (3.22)	2.87 (1.58)
	Organ failure	3	11.62 (3.73–36.25)	11.62 (28.83)	11.51 (4.44)	3.53 (2.07)
Hepatobiliary disorders	Jaundice	7	4.56 (2.17–9.58)	4.55 (19.35)	4.54 (2.44)	2.18 (1.16)
	Biliary obstruction	6	23.03 (10.26–51.68)	23.01 (123.9)	22.59 (11.48)	4.5 (3.39)
Infections and infestations	Anal abscess	5	7.3 (3.03–17.58)	7.29 (26.97)	7.25 (3.47)	2.86 (1.68)
Injury, poisoning, and procedural complications	Stoma site hemorrhage	6	29.19 (12.98–65.64)	29.16 (159.25)	28.48 (14.46)	4.83 (3.73)
Investigations	Blood pressure increased	173	11.8 (10.14–13.74)	11.49 (1645.38)	11.39 (10.03)	3.51 (3.29)
	Platelet count decreased	38	3.57 (2.59–4.92)	3.55 (69.69)	3.55 (2.72)	1.83 (1.36)
	Blood bilirubin increased	15	8.03 (4.83–13.35)	8.01 (91.47)	7.97 (5.21)	2.99 (2.27)
	Blood urine present	12	8.59 (4.87–15.17)	8.58 (79.79)	8.52 (5.3)	3.09 (2.29)
	Blood pressure abnormal	12	5.24 (2.97–9.25)	5.23 (40.93)	5.21 (3.24)	2.38 (1.58)
	Carcinoembryonic antigen increased	10	39.56 (21.06–74.33)	39.5 (363.12)	38.25 (22.57)	5.26 (4.37)
	Blood potassium decreased	9	3.66 (1.9–7.04)	3.65 (17.3)	3.65 (2.11)	1.87 (0.95)
	Ammonia increased	5	16.19 (6.7–39.15)	16.18 (70.24)	15.97 (7.63)	4 (2.81)
	Tumor marker increased	4	5.53 (2.07–14.77)	5.53 (14.76)	5.5 (2.42)	2.46 (1.17)
	Urine output decreased	4	7.1 (2.66–18.97)	7.09 (20.81)	7.06 (3.1)	2.82 (1.52)
	Blood albumin decreased	4	6.9 (2.58–18.44)	6.9 (20.05)	6.86 (3.01)	2.78 (1.48)
	Protein urine present	4	8.47 (3.17–22.66)	8.47 (26.15)	8.41 (3.69)	3.07 (1.78)
	Blood thyroid‐stimulating hormone increased	4	5.16 (1.93–13.78)	5.16 (13.35)	5.14 (2.26)	2.36 (1.07)
Metabolism and nutrition disorders	Decreased appetite	127	5.41 (4.54–6.46)	5.32 (445.44)	5.3 (4.58)	2.41 (2.15)
	Dehydration	39	3.96 (2.89–5.43)	3.94 (85.46)	3.93 (3.02)	1.98 (1.52)
	Hypophagia	16	6.84 (4.18–11.2)	6.83 (79.18)	6.8 (4.5)	2.76 (2.06)
Nervous system disorders	Neuropathy peripheral	44	4.45 (3.31–5.99)	4.43 (116.43)	4.41 (3.44)	2.14 (1.71)
	Hypersomnia	11	4.22 (2.33–7.63)	4.21 (26.85)	4.2 (2.56)	2.07 (1.23)
	Posterior reversible encephalopathy syndrome	9	8.77 (4.55–16.91)	8.76 (61.45)	8.71 (5.03)	3.12 (2.21)
Renal and urinary disorders	Proteinuria	30	14.38 (10.02–20.62)	14.31 (367.07)	14.15 (10.46)	3.82 (3.3)
	Renal impairment	24	2.99 (2–4.46)	2.98 (31.51)	2.97 (2.13)	1.57 (0.99)
	Nephrotic syndrome	12	19.86 (11.22–35.15)	19.82 (210.95)	19.51 (12.1)	4.29 (3.48)
	Chromaturia	7	4.51 (2.15–9.49)	4.51 (19.06)	4.5 (2.42)	2.17 (1.15)
	Renal‐limited thrombotic microangiopathy	3	44.31 (13.99–140.35)	44.29 (122.35)	42.72 (16.28)	5.42 (3.94)
Respiratory, thoracic, and mediastinal disorders	Dysphonia	123	21.82 (18.22–26.12)	21.39 (2350.61)	21.03 (18.09)	4.39 (4.13)
	Epistaxis	21	3.44 (2.24–5.28)	3.43 (36.11)	3.42 (2.39)	1.78 (1.16)
	Aphonia	17	10.75 (6.67–17.35)	10.73 (148.6)	10.64 (7.13)	3.41 (2.73)
	Pulmonary hemorrhage	3	6.32 (2.03–19.66)	6.32 (13.35)	6.29 (2.43)	2.65 (1.2)
Skin and subcutaneous tissue disorders	Palmar‐plantar erythrodysesthesia syndrome	51	23.67 (17.92–31.26)	23.47 (1076.33)	23.04 (18.25)	4.53 (4.12)
	Blister	27	4.59 (3.14–6.7)	4.57 (75.13)	4.56 (3.32)	2.19 (1.64)
	Skin ulcer	8	3.76 (1.88–7.52)	3.75 (16.1)	3.74 (2.09)	1.9 (0.94)
	Hyperkeratosis	6	11.63 (5.2–25.99)	11.62 (57.65)	11.51 (5.87)	3.53 (2.43)
Vascular disorders	Hypertension	53	2.64 (2.02–3.46)	2.63 (53.54)	2.62 (2.09)	1.39 (1)

### 3.4 Subgroup Information

Tables S1–S4 present the subgroup analyses of fruquintinib‐associated AEs across different demographic variables. Gender‐stratified analysis revealed fatigue as the most commonly reported AE (excluding death) in both sexes (Figure S1). Male participants were more susceptible to dysphonia, while female participants were more predisposed to increased blood pressure, diarrhea, nausea, and pain. Both age groups predominantly experienced fatigue. However, age‐specific differences were observed: patients aged 18–64 years had a relatively greater likelihood of myelosuppression and dysphonia, while those over 65 years were more susceptible to asthenia (Figure S2).

### 3.5 Onset Time of Events

**FIGURE 5:**
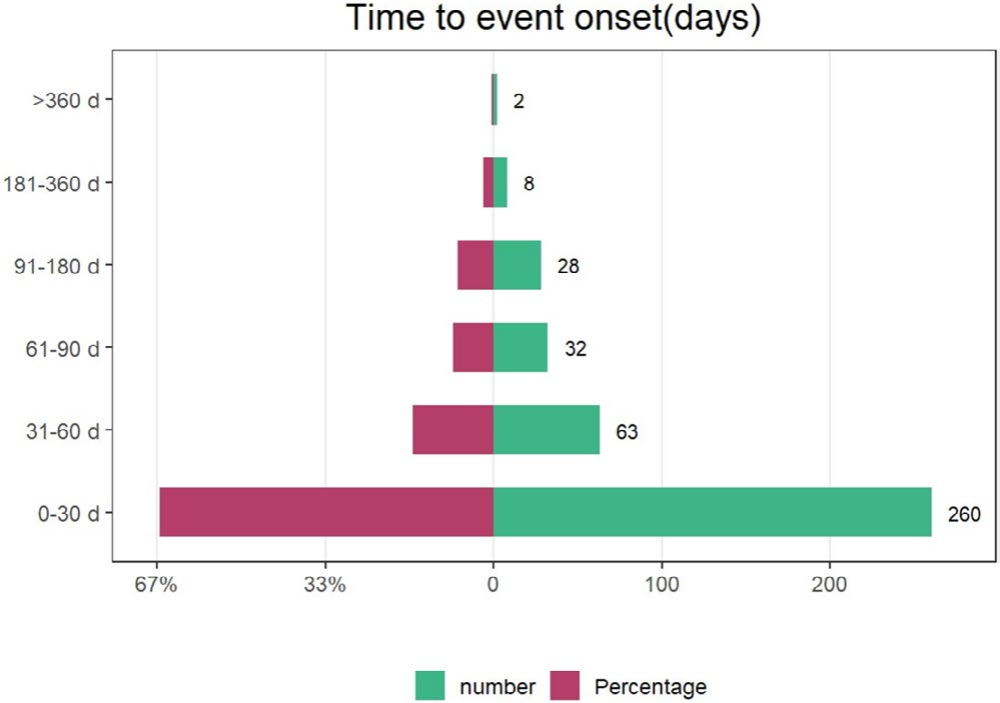
Onset time of adverse reactions related to fruquintinib.

We extracted onset times for fruquintinib‐associated AEs from the database. Following quality control measures that excluded erroneous reports, we analyzed 393 AEs with documented onset times. The Weibull distribution model (Table 5) revealed a distinct early‐onset failure pattern, with a median AE onset at 18 days postexposure (interquartile range [IQR] 7–46 days). Temporal analysis (Figure 5) revealed that 66.2% of fruquintinib‐associated AEs (*n* = 260) occurred within the initial treatment month, with the reporting frequency declining progressively thereafter. Importantly, clinicians should remain vigilant for both persistent and newly emerging AEs even after 1 year of continuous therapy.

**TABLE 5:** Time to onset of fruquintinib‐associated AEs and Weibull distribution analysis.

			Weibull distribution			
Cases	Median(IQR)	Min‐Max	Scale parameter: α 95% CI	Shape parameter: β 95% CI		Failure type
393	18(7,46)	1–693	32.74 28.49–36.99	0.81 0.75–0.87		Early failure

## 4 Discussion

Current studies on fruquintinib have focused predominantly on mechanistic and pharmacological investigations [27], clinical trial outcomes [28, 29], and comparative analyses with regorafenib or TAS‐102 [30]. Although case reports have documented rare adverse reactions [31, 32], real‐world safety data remain notably limited. To address this gap, we employed disproportionality analysis of FAERS data encompassing the postmarketing period following fruquintinib's FDA approval in late 2023. This study aims to identify clinically significant safety signals, with the ultimate goal of optimizing medication safety profiles in therapeutic practice.

The AEs of fruquintinib were observed more frequently in male patients (51.9%) than in female patients (41.1%), potentially reflecting the higher incidence of colorectal cancer (CRC) and consequently greater drug exposure in male populations. Sex‐specific patterns characterize CRC epidemiology, as evidenced by global surveillance data, which show a higher incidence in males than in females, attributable to biological determinants, genetic predispositions, and lifestyle factors [33, 34, 35]. Additionally, a greater proportion of AEs were observed in individuals aged 18–65 years (24.2%), while patients aged 65–85 years accounted for 19.6%. Contemporary cancer registry analyses account for this pharmacoepidemiological pattern, where the incidence of CRC has declined in adults over 50 years of age due to enhanced screening and improved risk factor management, while it shows a concerning rise in younger populations [1, 36, 37]. Geographically, the United States contributed the majority of reports (62.9%) to the surveillance system, significantly outpacing China (14.9%). However, China bears the heaviest burden of CRC, with both incidence and mortality rates exceeding global averages [38]. Therefore, these data highlight the critical need for intensified surveillance in China, with a particular emphasis on medical professionals, to optimize adverse event tracking for fruquintinib therapy. Notably, consumer reports constituted the highest proportion of AE signals. This phenomenon may reflect both heightened public health awareness and the convenience of the oral administration of fruquintinib, which reduces the need for frequent clinical visits.

Our disproportionality analyses revealed statistically significant SOC signals—neoplasms, benign, malignant, and unspecified (including cysts and polyps)—while general disorders and administration site conditions constituted the most frequently reported SOC category. The former included, but was not limited to, cancer pain, metastasis, or disease progression. These events are more likely attributable to the natural history of patients' advanced malignancies rather than direct fruquintinib toxicity. Table 4 summarizes the most frequently reported AEs—blood pressure increased, PPES, dysphonia, stomatitis, decreased appetite, fatigue, asthenia, abdominal pain upper, and myelosuppression—aligned with both the prescribing information and the safety data reported in the FRESCO phase III clinical trial data [8, 9]. This consistency reinforces the manageable safety profile of fruquintinib in real‐world clinical practice.

As a potent inhibitor of VEGFR‐1, –2, and –3, fruquintinib is a predictable hypertension signal reflecting its antiangiogenic pharmacology. In a Phase IV study evaluating the safety of fruquintinib in Chinese patients, the overall incidence of hypertension was 16% (6.6% of cases were Grade 3 or 4) [39]. In the FRESCO‐2 study, hypertension emerged as the most common treatment‐emergent AE, with an incidence of all‐grade hypertension of up to 37% and Grade 3 or worse hypertension in 14% of patients [8]. Harmonized analysis of three real‐world cohorts demonstrated that fruquintinib was more prone to Grade ≥ 3 hypertension than regorafenib, potentially reflecting its greater selectivity for VEGFR targets [40, 41, 42]. Current evidence from animal studies has demonstrated that under high‐salt conditions, the VEGFR2 kinase activity inhibitor fruquintinib uniquely blocks the adenosine A2A receptor (A2AR)‐mediated dermal lymphangiogenesis and elevates blood pressure [43]. In summary, these findings underscore the critical importance of implementing standardized hypertension monitoring protocols during fruquintinib therapy, with temporary drug discontinuation and initiation of antihypertensive therapy for Grade ≥ 3 hypertension.

In a Chinese postmarketing safety study of fruquintinib, PPES emerged as the most frequently reported AE, occurring in 19.8% of participants [39]. This condition typically begins with paresthesia and tingling, progresses to painful swelling, and in severe cases, is followed by blistering, desquamation, and ulceration [44]. The precise mechanisms underlying PPES remain incompletely understood. Current evidence suggests that concurrent inhibition of platelet‐derived growth factor receptor (PDGFR) and VEGFR signaling may impair physiological vascular repair mechanisms, which manifest most prominently in anatomical regions subjected to mechanical stress and repetitive trauma (e.g., palms and soles) [45, 46].

Dysphonia, though well documented in clinical trials [8], has remained frequently underrecognized in clinical practice and has rarely been addressed in therapeutic guidelines [12]. Hart et al. [47] demonstrated that VEGF/VEGFR inhibition‐induced vocal dysfunction stems from reduced microvascular density in the mucosa and in the underlying lamina propria, where capillary networks are essential for proper mucosal vibration during phonation. They also reported that aberrant vascular structures (e.g., telangiectasia) distort mucosal wave symmetry through localized mass effects on vocal fold tissues, which may also account for dysphonia [48]. Although dysphonia is reversible following drug withdrawal in most reported cases and is nonlife‐threatening, clinicians should emphasize this adverse reaction, as it may impose an impact on patients' quality of life [49].

Our disproportionality analysis revealed several unexpected safety signals, including peripheral neuropathy, dehydration, renal‐limited TMA, and PRES. A meta‐analysis demonstrated a significant association between VEGFR‐TKI therapy and increased risk of peripheral neuropathy [50], potentially attributable to the neuroprotective effects of VEGF in promoting axonal growth in dorsal root ganglia (DRG) and protecting DRG neurons from neuronal stress [51, 52]. We hypothesize that dehydration may result from either gastrointestinal toxicity (e.g., diarrhea and vomiting) or inadequate fluid intake (e.g., decreased appetite, mucositis), which are established AEs of fruquintinib therapy. A prior case report documented fruquintinib‐induced TMA [53]. Mechanistic studies revealed that VEGF signaling plays critical roles in maintaining podocyte integrity and function. Severe inhibition of this pathway can trigger podocyte effacement and inflammatory cascades, potentially progressing to nephrotic syndrome (encompassing TMA). These findings provide important insights for the early detection and prompt management of fruquintinib‐associated nephrotoxicity. Furthermore, several cases of fruquintinib‐associated PRES have been documented in the literature [14, 15]. Clinicians should maintain heightened vigilance for this rare but potentially life‐threatening complication in patients presenting with acute neurological deterioration. Notably, significant signals, such as ascites, hypersomnia, chromaturia, biliary obstruction, anal abscess, increased ammonia, decreased urine output, and large intestinal obstruction, are lacking in the literature. The pathogenic mechanisms underlying these AEs warrant further investigation through targeted clinical studies.

Our analysis revealed a median onset time of 18 days for fruquintinib‐associated AEs, with the majority (66.16%, *n* = 260) emerging within the first month and a 90.3% cumulative incidence occurring within 3 months. This distinct temporal distribution underscores the critical need for intensive AE monitoring during the initial treatment phase, particularly the first month, as early detection and prompt therapeutic adjustments are critical to ensure treatment safety and continuity.

This study systematically evaluated fruquintinib‐associated AEs using the FAERS database with a focused evaluation of blood pressure increased, PPES, and dysphonia, alongside other clinically significant signals. While the FAERS serves as the largest publicly accessible spontaneous reporting database for postmarketing surveillance, its inherent limitations must be acknowledged. The reliance on voluntary submissions leads to nonsystematic data collection and substantial underreporting, making it challenging to ascertain the true incidence of adverse events. Furthermore, the quality and completeness of reports are highly variable, often featuring missing baseline characteristics, undocumented concomitant medications and potential regional reporting biases, all of which may influence signal interpretation and complicate adjustment for potential confounders. Additionally, significant heterogeneity in drug dosage documentation and a high prevalence of missing dosage data in this dataset precluded a reliable analysis of dose–adverse event relationships. Consequently, the findings of this study should be interpreted as hypothesis‐generating, indicating statistical associations rather than causation. Future prospective studies or well‐curated electronic health record‐based cohorts with standardized data collection are warranted to validate these signals and elucidate potential causation or dose–response relationships. Given its recent clinical introduction, broader patient exposure may uncover additional safety signals. However, the current systematic identification of both anticipated and unanticipated adverse events enables evidence‐based risk mitigation and guides future mechanistic studies of this novel VEGFR inhibitor.

## 5 Conclusion

Our systematic analysis of FAERS data characterized the temporal profile and spectrum of fruquintinib‐associated AEs, confirming the expected blood pressure increase, palmar‐plantar erythrodysesthesia syndrome, dysphonia, and gastrointestinal toxicity, while novel signals, including peripheral neuropathy, dehydration, renal‐limited thrombotic microangiopathy, and posterior reversible encephalopathy syndrome, were identified. These findings not only validate clinical trial observations but also reveal previously underrecognized risks requiring enhanced monitoring, particularly during the first treatment month. The detection of these postmarketing safety signals underscores the need for both mechanistic studies to elucidate their pathogenesis and prospective studies to assess their clinical impact, ultimately guiding optimized risk mitigation strategies for this VEGFR inhibitor.

## Author Contributions

Yajing Xu and Yuyan Xu conceived and designed the study; Dongchen Wang performed the software operation; Yajing Xu, Dongchen Wang performed validation and formal analysis; all authors participated in the data curation; Yajing Xu, Dongchen Wang drafted the paper; Yuyan Xu participated in the review and editing of the paper; Dongchen Wang visualized the data; Yajing Xu performed supervision; all authors have read and agreed to the published version of the manuscript.

## Conflicts of Interest

The authors declare no conflicts of interest.

## Supporting information


**Figure S1:** Gender subgroup analysis of Fruquintinib‐associated AEs in the FAERS database, comparing AE occurrences between male and female patients.

**Figure S2:** Age subgroup analysis of Fruquintinib‐associated AEs in the FAERS database, comparing AE occurrences between patients aged 18–64 years and those aged ≥ 65 years.

**Table S1:** Signal strength of Fruquintinib‐associated AEs at the PT level in female patients from FAERS data.

**Table S2:** Signal strength of Fruquintinib‐associated AEs at the PT level in male patients from FAERS data.

**Table S3:** Signal strength of Fruquintinib‐associated AEs at the PT level in reported cases aged 18–64 years from FAERS data.

**Table S4:** Signal strength of Fruquintinib‐associated AEs at the PT level in reported cases aged ≥ 65 years from FAERS data.

## Data Availability

Research data are not shared.

## References

[1] R. L. Siegel , T. B. Kratzer , A. N. Giaquinto , H. Sung , and A. Jemal , “Cancer Statistics, 2025,” CA: Cancer Journal for Clinicians 75, no. 1 (2025): 10–45. 10.3322/caac.21871 PMC11745215 39817679

[2] R. S. Zheng , R. Chen , B. F. Han , et al., “Cancer Incidence and Mortality in China, 2022,” Zhonghua Zhong Liu Za Zhi 46, no. 3 (2024): 221–231. 38468501 10.3760/cma.j.cn112152-20240119-00035

[3] R. L. Siegel , N. S. Wagle , A. Cercek , R. A. Smith , and A. Jemal , “Colorectal Cancer Statistics, 2023,” CA: Cancer Journal for Clinicians 73, no. 3 (2023): 233–254. 10.3322/caac.21772 36856579

[4] R. J. Mayer , E. Van Cutsem , A. Falcone , et al., “Randomized Trial of TAS‐102 for Refractory Metastatic Colorectal Cancer,” New England Journal of Medicine 372, no. 20 (2015): 1909–1919. 25970050 10.1056/NEJMoa1414325

[5] A. Grothey , E. Van Cutsem , A. Sobrero , et al., “Regorafenib Monotherapy for Previously Treated Metastatic Colorectal Cancer (CORRECT): An International, Multicentre, Randomised, Placebo‐Controlled, Phase 3 Trial,” Lancet 381, no. 9863 (2013): 303–312. 23177514 10.1016/S0140-6736(12)61900-X

[6] Q. Sun , J. Zhou , Z. Zhang , et al., “Discovery of Fruquintinib, a Potent and Highly Selective Small Molecule Inhibitor of VEGFR 1, 2, 3 Tyrosine Kinases for Cancer Therapy,” Cancer Biology & Therapy 15, no. 12 (2014): 1635–1645. 25482937 10.4161/15384047.2014.964087 PMC4622458

[7] Y. Gu , J. Wang , K. Li , et al., “Preclinical Pharmacokinetics and Disposition of a Novel Selective VEGFR Inhibitor Fruquintinib (HMPL‐013) and the Prediction of Its Human Pharmacokinetics,” Cancer Chemotherapy and Pharmacology 74, no. 1 (2014): 95–115. 24817647 10.1007/s00280-014-2471-3

[8] A. Dasari , S. Lonardi , R. Garcia‐Carbonero , et al., “Fruquintinib Versus Placebo in Patients With Refractory Metastatic Colorectal Cancer (FRESCO‐2): An International, Multicentre, Randomised, Double‐Blind, Phase 3 Study,” Lancet 402, no. 10395 (2023): 41–53. 37331369 10.1016/S0140-6736(23)00772-9

[9] J. Li , S. Qin , R. H. Xu , et al., “Effect of Fruquintinib vs Placebo on Overall Survival in Patients With Previously Treated Metastatic Colorectal Cancer: The FRESCO Randomized Clinical Trial,” JAMA 319, no. 24 (2018): 2486–2496. 29946728 10.1001/jama.2018.7855 PMC6583690

[10] X. Wu , J. Wang , D. Wang , et al., “Fruquintinib Plus Sintilimab in Treated Advanced Endometrial Cancer (EMC) Patients (Pts) With PMMR Status: Results From a Multicenter, Single‐Arm Phase 2 Study,” Journal of Clinical Oncology 42, no. 16 (2024): 5619 5619.

[11] F. Wang , L. Shen , W. Guo , et al., “Fruquintinib Plus Paclitaxel Versus Placebo Plus Paclitaxel for Gastric or Gastroesophageal Junction Adenocarcinoma: The Randomized Phase 3 FRUTIGA Trial,” Nature Medicine 30, no. 8 (2024): 2189–2198. 10.1038/s41591-024-02989-6 38824242

[12] C. Eng , A. Dasari , S. Lonardi , et al., “Fruquintinib Versus Placebo in Patients With Refractory Metastatic Colorectal Cancer: Safety Analysis of FRESCO‐2,” Oncologist 30, no. 3 (2025): oyae360. 40163688 10.1093/oncolo/oyae360 PMC11957243

[13] “Fruquintinib, LiverTox: Clinical and Research Information on Drug‐Induced Liver Injury,” (2012). 31643176

[14] C. B. Ledet , U. Sener , D. R. Johnson , K. Ku , and T. R. Halfdanarson , “Fruquintinib‐Associated Posterior Reversible Encephalopathy Syndrome in a Patient With Multiply Metastatic Rectal Cancer,” Clinical Colorectal Cancer 24, no. 1 (2025): 98–100. 39343651 10.1016/j.clcc.2024.08.006

[15] L. Wang , Z. Zeng , and Z. Wu , “Case Report: PRES Associated With Fruquintinib in a Patient With Metastatic Colon Cancer,” Neurological Sciences 44, no. 11 (2023): 4111–4114. 37581770 10.1007/s10072-023-06991-7 PMC10570160

[16] C. Michel , E. Scosyrev , M. Petrin , and R. Schmouder , “Can Disproportionality Analysis of Post Marketing Case Reports Be Used for Comparison of Drug Safety Profiles?,” Clinical Drug Investigation 37, no. 5 (2017): 415–422. 28224371 10.1007/s40261-017-0503-6

[17] M. M. Dhodapkar , X. Shi , R. Ramachandran , E. M. Chen , J. D. Wallach , and J. S. Ross , “Characterization and Corroboration of Safety Signals Identified From the US Food and Drug Administration Adverse Event Reporting System, 2008‐19: Cross Sectional Study,” BMJ 379 (2022): e071752. 36198428 10.1136/bmj-2022-071752 PMC9533298

[18] M. Alomar , A. M. Tawfiq , N. Hassan , and S. Palaian , “Post Marketing Surveillance of Suspected Adverse Drug Reactions Through Spontaneous Reporting: Current Status, Challenges and the Future,” Therapeutic Advances in Drug Safety 11 (2020): 2042098620938595. 32843958 10.1177/2042098620938595 PMC7418468

[19] B. Zhao , X. Zhang , M. Chen , and Y. Wang , “A Real‐World Data Analysis of Acetylsalicylic Acid in FDA Adverse Event Reporting System (FAERS) Database,” Expert Opinion on Drug Metabolism & Toxicology 19, no. 6 (2023): 381–387. 37421631 10.1080/17425255.2023.2235267

[20] Y. Wang , B. Zhao , H. Yang , and Z. Wan , “A Real‐World Pharmacovigilance Study of FDA Adverse Event Reporting System Events for Sildenafil,” Andrology 12, no. 4 (2024): 785–792. 37724699 10.1111/andr.13533

[21] C. Chen , B. Wu , C. Zhang , and T. Xu , “Immune‐Related Adverse Events Associated With Immune Checkpoint Inhibitors: An Updated Comprehensive Disproportionality Analysis of the FDA Adverse Event Reporting System,” International Immunopharmacology 95 (2021): 107498. 33725634 10.1016/j.intimp.2021.107498

[22] C. Zhong , Q. Zheng , B. Zhao , and T. Ren , “A Real‐World Pharmacovigilance Study Using Disproportionality Analysis of United States Food and Drug Administration Adverse Event Reporting System Events for Vinca Alkaloids: Comparing Vinorelbine and Vincristine,” Expert Opinion on Drug Safety 23, no. 11 (2024): 1427–1437. 39340205 10.1080/14740338.2024.2410436

[23] B. Zhao , Y. Fu , S. Cui , X. Chen , S. Liu , and L. Luo , “A Real‐World Disproportionality Analysis of Everolimus: Data Mining of the Public Version of FDA Adverse Event Reporting System,” Frontiers in Pharmacology 15 (2024): 1333662. 38533254 10.3389/fphar.2024.1333662 PMC10964017

[24] H. Yang , Z. Wan , M. Chen , X. Zhang , W. Cui , and B. Zhao , “A Real‐World Data Analysis of Topotecan in the FDA Adverse Event Reporting System (FAERS) Database,” Expert Opinion on Drug Metabolism & Toxicology 19, no. 4 (2023): 217–223. 37243615 10.1080/17425255.2023.2219390

[25] M. Lindquist , M. Stahl , A. Bate , I. R. Edwards , and R. H. Meyboom , “A Retrospective Evaluation of a Data Mining Approach to Aid Finding New Adverse Drug Reaction Signals in the WHO International Database,” Drug Safety 23, no. 6 (2000): 533–542. 11144660 10.2165/00002018-200023060-00004

[26] T. Sakaeda , A. Tamon , K. Kadoyama , and Y. Okuno , “Data Mining of the Public Version of the FDA Adverse Event Reporting System,” International Journal of Medical Sciences 10, no. 7 (2013): 796–803. 23794943 10.7150/ijms.6048 PMC3689877

[27] Q. Song , H. Wu , Y. Jin , et al., “Fruquintinib Inhibits the Migration and Invasion of Colorectal Cancer Cells by Modulating Epithelial‐Mesenchymal Transition via TGF‐beta/Smad Signaling Pathway,” Frontiers in Oncology 15 (2025): 1503133. 40134588 10.3389/fonc.2025.1503133 PMC11932892

[28] X. Zhang , T. Zhao , B. Zheng , et al., “Fruquintinib as First‐Line or Second‐Line Treatment in Unresectable or Metastatic Soft‐Tissue Sarcoma: A Prospective, Single‐Arm Phase II Study,” Clinical and Translational Medicine 15, no. 4 (2025): e70308. 40235094 10.1002/ctm2.70308 PMC12000221

[29] M. Cheng , M. Jin , S. Yang , et al., “Effect of Radiotherapy Exposure on Fruquintinib Plus Sintilimab Treatment in Refractory Microsatellite Stable Metastatic Colorectal Cancer: A Prospective Observation Study,” Journal for Immunotherapy of Cancer 13, no. 1 (2025): e009415. 39755582 10.1136/jitc-2024-009415 PMC11749590

[30] Q. Zhang , Q. Wang , X. Wang , J. Li , L. Shen , and Z. Peng , “Regorafenib, TAS‐102, or Fruquintinib for Metastatic Colorectal Cancer: Any Difference in Randomized Trials?,” International Journal of Colorectal Disease 35, no. 2 (2020): 295–306. 31848739 10.1007/s00384-019-03477-x

[31] Q. Liu , Q. Wang , H. Tan , et al., “Renal‐Limited Thrombotic Microangiopathy Induced by Fruquintinib and Tislelizumab: A Case Report,” Nephrology (Carlton) 30, no. 6 (2025): e70067. 40528285 10.1111/nep.70067

[32] E. Hafliger , R. Nili‐Asgari , J. Netter , and J. Taieb , “Case Report of Two Fatal Cerebral Hemorrhages in Patients With Metastatic Colorectal Cancer Treated With Fruquintinib,” European Journal of Cancer 224 (2025): 115517. 40410102 10.1016/j.ejca.2025.115517

[33] T. Torres , E. Arellano Villanueva , Y. Alsabawi , D. Fofana , and M. K. Tripathi , “Unraveling Early Onset Disparities and Determinants: An Analysis of Colorectal Cancer Outcomes and Trends in Texas,” Cureus 17, no. 4 (2025): e83124. 40438851 10.7759/cureus.83124 PMC12119150

[34] P. N. Abotchie , S. W. Vernon , and X. L. Du , “Gender Differences in Colorectal Cancer Incidence in the United States, 1975‐2006,” Journal of Women's Health (2002) 21, no. 4 (2012): 393–400. 10.1089/jwh.2011.2992 PMC3321677 22149014

[35] A. White , L. Ironmonger , R. J. C. Steele , N. Ormiston‐Smith , C. Crawford , and A. Seims , “A Review of Sex‐Related Differences in Colorectal Cancer Incidence, Screening Uptake, Routes to Diagnosis, Cancer Stage and Survival in the UK,” BMC Cancer 18, no. 1 (2018): 906. 30236083 10.1186/s12885-018-4786-7 PMC6149054

[36] H. Kim , A. Melio , V. Simianu , and G. Mankaney , “Challenges and Opportunities for Colorectal Cancer Prevention in Young Patients,” Cancers (Basel) 17, no. 12 (2025): 2043. 40563692 10.3390/cancers17122043 PMC12191148

[37] E. M. Stoffel and C. C. Murphy , “Epidemiology and Mechanisms of the Increasing Incidence of Colon and Rectal Cancers in Young Adults,” Gastroenterology 158, no. 2 (2020): 341–353. 31394082 10.1053/j.gastro.2019.07.055 PMC6957715

[38] J. J. Li , Y. M. Zhang , Y. T. Ji , et al., “Comparison Analyses of Global Burden of Colorectal Cancer,” Zhonghua Zhong Liu Za Zhi 47, no. 4 (2025): 308–315. 40268547 10.3760/cma.j.cn112152-20240308-00102

[39] J. Li , Z. Wang , H. Zhong , et al., “A Phase IV Study to Evaluate the Safety of Fruquintinib in Chinese Patients in Real‐World Clinical Practice,” Oncologist 29, no. 8 (2024): e1012–e1019. 38642091 10.1093/oncolo/oyae073 PMC11299944

[40] Q. Zhang , M. Chen , Z. Wang , et al., “Efficacy and Safety Comparison of Regorafenib and Fruquintinib in Metastatic Colorectal Cancer‐An Observational Cohort Study in the Real World,” Clinical Colorectal Cancer 21, no. 3 (2022): e152–e161. 35216918 10.1016/j.clcc.2022.01.007

[41] Y. Y. Deng , X. Y. Zhang , P. F. Zhu , et al., “Comparison of the Efficacy and Safety of Fruquintinib and Regorafenib in the Treatment of Metastatic Colorectal Cancer: A Real‐World Study,” Frontiers in Oncology 13 (2023): 1097911. 36937443 10.3389/fonc.2023.1097911 PMC10020225

[42] Y. Dai , L. Sun , L. Zhuang , et al., “Efficacy and Safety of Low‐Dose Apatinib Plus S‐1 Versus Regorafenib and Fruquintinib for Refractory Metastatic Colorectal Cancer: A Retrospective Cohort Study,” Journal of Gastrointestinal Oncology 13, no. 2 (2022): 722–731. 35557597 10.21037/jgo-22-285 PMC9086039

[43] T. Zhuang , Y. Lei , J. J. Chang , et al., “A2AR‐Mediated Lymphangiogenesis via VEGFR2 Signaling Prevents Salt‐Sensitive Hypertension,” European Heart Journal 44, no. 29 (2023): 2730–2742. 37377160 10.1093/eurheartj/ehad377 PMC10393074

[44] E. Nagore , A. Insa , and O. Sanmartin , “Antineoplastic Therapy‐Induced Palmar Plantar Erythrodysesthesia ('hand‐Foot') Syndrome. Incidence, Recognition and Management,” American Journal of Clinical Dermatology 1, no. 4 (2000): 225–234. 11702367 10.2165/00128071-200001040-00004

[45] C. Robert , J. C. Soria , A. Spatz , et al., “Cutaneous Side‐Effects of Kinase Inhibitors and Blocking Antibodies,” Lancet Oncology 6, no. 7 (2005): 491–500. 15992698 10.1016/S1470-2045(05)70243-6

[46] M. E. Lacouture , S. Wu , C. Robert , et al., “Evolving Strategies for the Management of Hand‐Foot Skin Reaction Associated With the Multitargeted Kinase Inhibitors Sorafenib and Sunitinib,” Oncologist 13, no. 9 (2008): 1001–1011. 18779536 10.1634/theoncologist.2008-0131

[47] D. M. Hartl , R. Bahleda , A. Hollebecque , J. Bosq , C. Massard , and J. C. Soria , “Bevacizumab‐Induced Laryngeal Necrosis,” Annals of Oncology 23, no. 1 (2012): 276–278. 22056850 10.1093/annonc/mdr515

[48] D. M. Hartl , C. Ferte , Y. Loriot , et al., “Dysphonia Induced by Vascular Endothelium Growth Factor/Vascular Endothelium Growth Factor Receptor Inhibitors,” Investigational New Drugs 28, no. 6 (2010): 884–886. 19756374 10.1007/s10637-009-9314-9

[49] E. Saavedra , A. Hollebecque , J. C. Soria , and D. M. Hartl , “Dysphonia Induced by Anti‐Angiogenic Compounds,” Investigational New Drugs 32, no. 4 (2014): 774–782. 24343672 10.1007/s10637-013-0049-2

[50] B. Roy , A. Das , K. Ashish , et al., “Neuropathy With Vascular Endothelial Growth Factor Receptor Tyrosine Kinase Inhibitors: A Meta‐Analysis,” Neurology 93, no. 2 (2019): e143–e148. 31167931 10.1212/WNL.0000000000007743

[51] C. Ruiz de Almodovar , D. Lambrechts , M. Mazzone , and P. Carmeliet , “Role and Therapeutic Potential of VEGF in the Nervous System,” Physiological Reviews 89, no. 2 (2009): 607–648. 19342615 10.1152/physrev.00031.2008

[52] A. Verheyen , E. Peeraer , R. Nuydens , et al., “Systemic Anti‐Vascular Endothelial Growth Factor Therapies Induce a Painful Sensory Neuropathy,” Brain 135, no. 9 (2012): 2629–2641. 22734125 10.1093/brain/aws145

[53] R. Zhao , R. Fan , Y. Pan , Y. Han , Y. Wang , and W. Chen , “Fruquintinib‐Induced Renal‐Limited Thrombotic Microangiopathy: A Case Report,” BMC Nephrology 25, no. 1 (2024): 170. 38762494 10.1186/s12882-024-03598-8 PMC11102188

