# Supplementary figures and images for: A Real‐World Pharmacovigilance Study of Fruquintinib Based on the FDA Adverse Event Reporting System (FAERS) Database
Source: Cancer Med. 2025 Nov 7;14(21):e71352. doi: 10.1002/cam4.71352 (PMC12593544; doi:10.1002/cam4.71352)

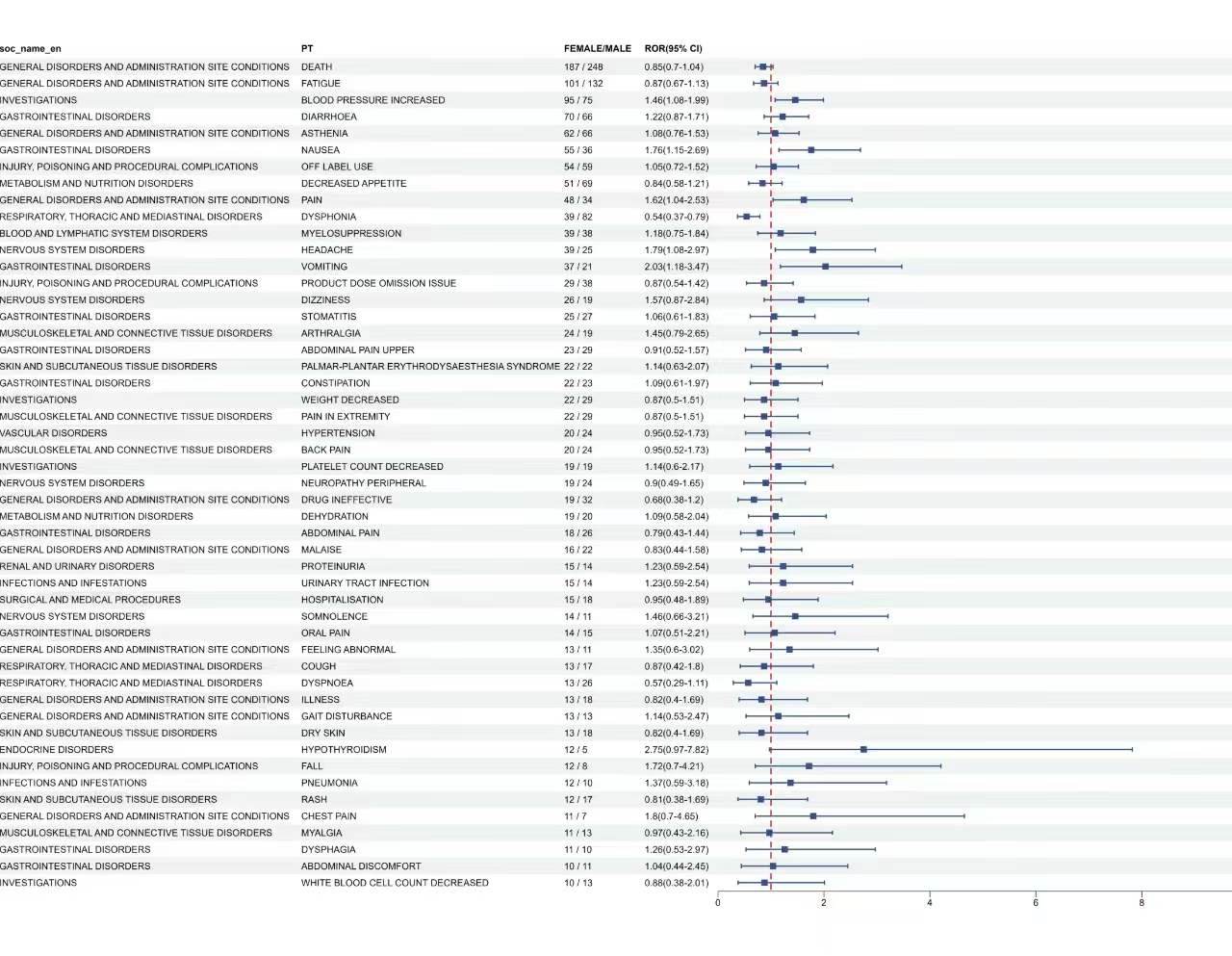

Supplement: Supplementary file 1 — Figure S1: Gender subgroup analysis of Fruquintinib‐associated AEs in the FAERS database, comparing AE occurrences between male and female patients. [file CAM4-14-e71352-s001.jpg]

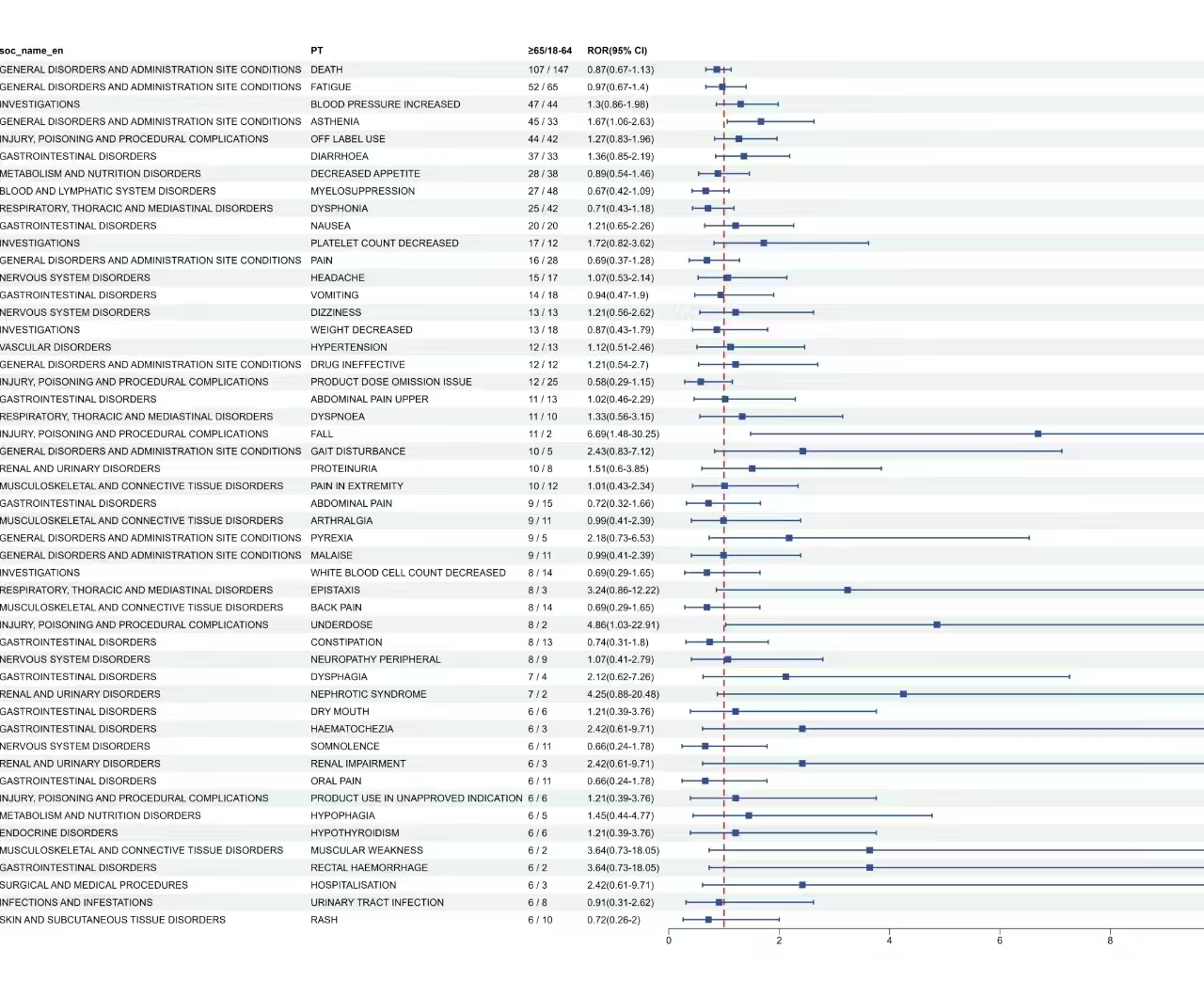

Supplement: Supplementary file 2 — Figure S2: Age subgroup analysis of Fruquintinib‐associated AEs in the FAERS database, comparing AE occurrences between patients aged 18–64 years and those aged ≥ 65 years. [file CAM4-14-e71352-s006.jpg]
