# Supplementary material for: A Real‐World Pharmacovigilance Study of Fruquintinib Based on the FDA Adverse Event Reporting System (FAERS) Database
Source: Cancer Med. 2025 Nov 7;14(21):e71352. doi: 10.1002/cam4.71352 (PMC12593544; doi:10.1002/cam4.71352)
Supplement: Supplementary file 3 — Table S1: Signal strength of Fruquintinib‐associated AEs at the PT level in female patients from FAERS data. [file CAM4-14-e71352-s005.docx]

Signal strength of Fruquintinib-associated adverse events at the PT level in female patients (FAERS)

| SOC | PTs | Cases | ROR (95%Cl) | PRR (χ2) | EBGM  (EBGM05) | IC  (IC025) |
| --- | --- | --- | --- | --- | --- | --- |
| Blood and lymphatic system disorders | Myelosuppression | 39 | 11.66 (8.49- 16.02) | 11.51 (371.64) | 11.42 (8.76) | 3.51 (3.05) |
| Endocrine disorders | Hypothyroidism | 12 | 7.29  (4.13-12.88) | 7.27 (64.54) | 7.23 (4.49) | 2.85 (2.05) |
| Gastrointestinal disorders | Stomatitis | 25 | 6.92  (4.66- 10.27) | 6.87 (124.9) | 6.84 (4.92) | 2.77 (2.2) |
| Gastrointestinal disorders | Oral pain | 14 | 14.97 (8.83- 25.39) | 14.9 (179.68) | 14.75 (9.48) | 3.88 (3.13) |
| Gastrointestinal disorders | Ascites | 6 | 6.63  (2.97- 14.8) | 6.62 (28.49) | 6.59 (3.37) | 2.72 (1.63) |
| Gastrointestinal disorders | Gingival pain | 5 | 16.19  (6.7- 39.13) | 16.16 (70.31) | 15.99 (7.64) | 4 (2.81) |
| Gastrointestinal disorders | Tongue ulceration | 4 | 30.83 (11.44- 83.1) | 30.79 (112.8) | 30.14 (13.15) | 4.91 (3.61) |
| Gastrointestinal disorders | Tongue discomfort | 3 | 11.69 (3.75- 36.43) | 11.67 (29.04) | 11.59 (4.47) | 3.53 (2.08) |
| Gastrointestinal disorders | Oral discomfort | 3 | 5.44  (1.75- 16.91) | 5.43 (10.82) | 5.42 (2.1) | 2.44 (0.99) |
| Gastrointestinal disorders | Proctalgia | 3 | 13.24 (4.24- 41.3) | 13.23 (33.59) | 13.11 (5.06) | 3.71 (2.26) |
| General disorders and administration site conditions | Death | 187 | 8.98  (7.73- 10.42) | 8.43 (1226.54) | 8.38 (7.4) | 3.07 (2.85) |
| General disorders and administration site conditions | Fatigue | 101 | 2.79 (2.28- 3.4) | 2.72 (111.22) | 2.72 (2.3) | 1.44 (1.15) |
| General disorders and administration site conditions | Asthenia | 62 | 4.15  (3.23- 5.34) | 4.08 (144.43) | 4.07 (3.29) | 2.02 (1.66) |
| Infections and infestations | Kidney infection | 5 | 4.27  (1.78- 10.29) | 4.27 (12.48) | 4.26 (2.04) | 2.09 (0.91) |
| Investigations | Blood pressure increased | 95 | 15.1 (12.29- 18.55) | 14.6 (1194.24) | 14.46 (12.18) | 3.85 (3.55) |
| Investigations | Platelet count decreased | 19 | 4.44 (2.83- 6.98) | 4.42 (50.18) | 4.41 (3.02) | 2.14 (1.49) |
| Investigations | Blood pressure abnormal | 8 | 7.44 (3.71- 14.92) | 7.42 (44.22) | 7.39 (4.13) | 2.88 (1.92) |
| Investigations | Blood potassium decreased | 7 | 5.21 (2.48- 10.95) | 5.2 (23.64) | 5.18 (2.78) | 2.37 (1.35) |
| Investigations | Carcinoembryonic antigen increased | 6 | 43.4 (19.24- 97.9) | 43.31 (240.54) | 42.04 (21.28) | 5.39 (4.28) |
| Investigations | Blood bilirubin increased | 6 | 9.41 (4.21- 21.02) | 9.39 (44.7) | 9.34 (4.77) | 3.22 (2.13) |
| Investigations | Urine output decreased | 4 | 16.66 (6.21- 44.68) | 16.63 (58.09) | 16.45 (7.21) | 4.04 (2.74) |
| Investigations | Blood albumin decreased | 4 | 18.85 (7.02- 50.6) | 18.83 (66.62) | 18.59 (8.14) | 4.22 (2.91) |
| Investigations | Blood urine present | 4 | 7.76  (2.9- 20.75) | 7.75 (23.39) | 7.71 (3.39) | 2.95 (1.65) |
| Metabolism and nutrition disorders | Decreased appetite | 51 | 4.73 (3.59- 6.25) | 4.66 (146.81) | 4.65 (3.69) | 2.22 (1.81) |
| Metabolism and nutrition disorders | Dehydration | 19 | 4.12 (2.62- 6.48) | 4.1 (44.5) | 4.09 (2.8) | 2.03 (1.38) |
| Metabolism and nutrition disorders | Hypophagia | 5 | 4.53 (1.88- 10.9) | 4.52 (13.67) | 4.51 (2.16) | 2.17 (0.99) |
| Nervous system disorders | Neuropathy peripheral | 19 | 4.74 (3.02 - 7.45) | 4.71 (55.46) | 4.7 (3.22) | 2.23 (1.58) |
| Nervous system disorders | Cerebral haemorrhage | 5 | 5.71 (2.37- 13.76) | 5.71 (19.33) | 5.69 (2.72) | 2.51 (1.33) |
| Nervous system disorders | Posterior reversible encephalopathy syndrome | 4 | 7.9 (2.96 - 21.13) | 7.89 (23.95) | 7.85 (3.45) | 2.97 (1.68) |
| Product issues | Product physical issue | 4 | 5.49 (2.06- 14.67) | 5.48 (14.62) | 5.47 (2.4) | 2.45 (1.16) |
| Renal and urinary disorders | Proteinuria | 15 | 21.39 (12.83- 35.67) | 21.28 (285.63) | 20.98 (13.67) | 4.39 (3.66) |
| Renal and urinary disorders | Renal impairment | 10 | 3.76  (2.02- 7.01) | 3.75 (20.15) | 3.74 (2.23) | 1.9 (1.03) |
| Renal and urinary disorders | Nephrotic syndrome | 7 | 36.48 (17.21- 77.33) | 36.39 (234.8) | 35.49 (18.93) | 5.15 (4.11) |
| Renal and urinary disorders | Chromaturia | 4 | 5.93  (2.22- 15.84) | 5.92 (16.3) | 5.9 (2.59) | 2.56 (1.27) |
| Respiratory, thoracic and mediastinal disorders | Dysphonia | 39 | 13.95 (10.15- 19.16) | 13.76 (457.4) | 13.63 (10.45) | 3.77 (3.31) |
| Respiratory, thoracic and mediastinal disorders | Epistaxis | 9 | 3.64  (1.89- 7) | 3.63 (17.1) | 3.62 (2.09) | 1.86 (0.94) |
| Respiratory, thoracic and mediastinal disorders | Aphonia | 9 | 9.97  (5.17- 19.22) | 9.94 (71.84) | 9.87 (5.7) | 3.3 (2.39) |
| Skin and subcutaneous tissue disorders | Palmar-plantar erythrodysaesthesia syndrome | 22 | 27.56 (18.04- 42.09) | 27.34 (547.73) | 26.83 (18.83) | 4.75 (4.14) |
