# Supplementary material for: A Real‐World Pharmacovigilance Study of Fruquintinib Based on the FDA Adverse Event Reporting System (FAERS) Database
Source: Cancer Med. 2025 Nov 7;14(21):e71352. doi: 10.1002/cam4.71352 (PMC12593544; doi:10.1002/cam4.71352)
Supplement: Supplementary file 4 — Table S2: Signal strength of Fruquintinib‐associated AEs at the PT level in male patients from FAERS data. [file CAM4-14-e71352-s003.docx]

Signal strength of Fruquintinib-associated adverse events at the PT level in male patients (FAERS).

| SOC | | PTs | Cases | | ROR  (95%Cl) | PRR (χ2) | | EBGM(EBGM05) | | IC(IC025) | |
| --- | --- | --- | --- | --- | --- | --- | --- | --- | --- | --- | --- |
| Blood and lymphatic system disorders | Myelosuppression | | 38 | 8.83 (6.4 - 12.18) | | | 8.73 (257.46) | | 8.64 (6.6) | | 3.11 (2.64) |
| Gastrointestinal disorders | Abdominal pain upper | | 29 | 4.03 (2.8 - 5.82) | | | 4.01 (65.19) | | 3.99 (2.93) | | 2 (1.4) |
|  | Stomatitis | | 27 | 11.22 (7.66 - 16.43) | | | 11.13 (245.19) | | 10.97 (7.97) | | 3.46 (2.9) |
|  | Oral pain | | 15 | 18.26 (10.92 - 30.52) | | | 18.17 (237.39) | | 17.74 (11.54) | | 4.15 (3.42) |
|  | Dry mouth | | 12 | 4.56 (2.58 - 8.06) | | | 4.55 (33.05) | | 4.53 (2.81) | | 2.18 (1.37) |
|  | Rectal haemorrhage | | 11 | 5.46 (3.01 - 9.89) | | | 5.44 (39.64) | | 5.41 (3.29) | | 2.44 (1.6) |
|  | Ascites | | 9 | 5.97 (3.1 - 11.52) | | | 5.96 (36.84) | | 5.92 (3.41) | | 2.56 (1.65) |
|  | Glossodynia | | 4 | 11.69 (4.35 - 31.42) | | | 11.68 (38.41) | | 11.5 (5.03) | | 3.52 (2.22) |
|  | Tongue ulceration | | 3 | 29.06 (9.15 - 92.24) | | | 29.03 (77.99) | | 27.92 (10.62) | | 4.8 (3.32) |
|  | Large intestinal obstruction | | 3 | 20.01 (6.35 - 63.07) | | | 19.99 (52.64) | | 19.47 (7.45) | | 4.28 (2.81) |
| General disorders and administration site conditions | Death | | 248 | 4.94 (4.34 - 5.63) | | | 4.63 (712.62) | | 4.6 (4.13) | | 2.2 (2.01) |
|  | Fatigue | | 132 | 3.77 (3.16 - 4.49) | | | 3.65 (255.43) | | 3.63 (3.14) | | 1.86 (1.61) |
|  | Asthenia | | 66 | 3.68 (2.88 - 4.7) | | | 3.62 (125.3) | | 3.61 (2.94) | | 1.85 (1.49) |
|  | Mucosal inflammation | | 6 | 4.22 (1.89 - 9.42) | | | 4.21 (14.62) | | 4.19 (2.14) | | 2.07 (0.97) |
|  | Terminal state | | 3 | 6.2 (1.99 - 19.33) | | | 6.2 (12.96) | | 6.15 (2.38) | | 2.62 (1.17) |
|  | Organ failure | | 3 | 16.57 (5.27 - 52.09) | | | 16.56 (42.85) | | 16.2 (6.21) | | 4.02 (2.55) |
| Hepatobiliary disorders | Liver disorder | | 11 | 5.37 (2.96 - 9.73) | | | 5.35 (38.69) | | 5.32 (3.24) | | 2.41 (1.57) |
|  | Jaundice | | 5 | 4.6 (1.91- 11.09) | | | 4.59 (13.97) | | 4.57 (2.19) | | 2.19 (1.01) |
|  | Biliary obstruction | | 4 | 18.99 (7.03 - 51.28) | | | 18.96 (66.29) | | 18.49 (8.05) | | 4.21 (2.9) |
| Infections and infestations | Anal abscess | | 4 | 9.04 (3.37 - 24.25) | | | 9.03 (28.19) | | 8.93 (3.91) | | 3.16 (1.86) |
| Injury, poisoning and procedural complications | Stoma site haemorrhage | | 3 | 19.46 (6.18 - 61.31) | | | 19.44 (51.07) | | 18.95 (7.25) | | 4.24 (2.77) |
| Investigations | Blood pressure increased | | 75 | 10.05 (7.98 - 12.66) | | | 9.83 (588.49) | | 9.71 (8.01) | | 3.28 (2.94) |
|  | Platelet count decreased | | 19 | 2.99 (1.9- 4.7) | | | 2.98 (24.93) | | 2.97 (2.04) | | 1.57 (0.92) |
|  | Blood bilirubin increased | | 9 | 7.25 (3.76 - 13.99) | | | 7.23 (47.87) | | 7.17 (4.14) | | 2.84 (1.92) |
|  | Blood urine present | | 8 | 8.21 (4.08 - 16.49) | | | 8.19 (49.93) | | 8.11 (4.52) | | 3.02 (2.05) |
|  | Carcinoembryonic antigen increased | | 4 | 36.74 (13.44 - 100.45) | | | 36.69 (132.03) | | 34.93 (15.06) | | 5.13 (3.8) |
|  | Blood sodium decreased | | 4 | 4.9 (1.83 - 13.11) | | | 4.9 (12.32) | | 4.87 (2.14) | | 2.28 (0.99) |
|  | Tumour marker increased | | 3 | 24.38 (7.71 - 77.11) | | | 24.36 (64.96) | | 23.58 (9) | | 4.56 (3.08) |
|  | Ammonia increased | | 3 | 13.34 (4.25 - 41.83) | | | 13.33 (33.58) | | 13.1 (5.03) | | 3.71 (2.25) |
| Metabolism and nutrition disorders | Decreased appetite | | 69 | 5.52 (4.34 - 7.01) | | | 5.42 (247.58) | | 5.38 (4.4) | | 2.43 (2.08) |
|  | Dehydration | | 20 | 3.8 (2.45 - 5.91) | | | 3.79 (40.86) | | 3.77 (2.61) | | 1.92 (1.28) |
|  | Hypophagia | | 11 | 7.97 (4.39 - 14.45) | | | 7.94 (66.06) | | 7.87 (4.78) | | 2.98 (2.14) |
| Musculoskeletal and connective tissue disorders | Pain in extremity | | 29 | 2.84 (1.97 - 4.09) | | | 2.82 (34.04) | | 2.81 (2.07) | | 1.49 (0.96) |
|  | Bone pain | | 7 | 4.16 (1.97 - 8.74) | | | 4.15 (16.64) | | 4.13 (2.22) | | 2.05 (1.02) |
| Nervous system disorders | Neuropathy peripheral | | 24 | 4.16 (2.78 - 6.23) | | | 4.14 (56.88) | | 4.12 (2.94) | | 2.04 (1.46) |
|  | Hypersomnia | | 8 | 5.49 (2.74 - 11.02) | | | 5.48 (29.1) | | 5.45 (3.04) | | 2.45 (1.48) |
|  | Posterior reversible encephalopathy syndrome | | 4 | 7.95 (2.96 - 21.3) | | | 7.94 (23.99) | | 7.86 (3.44) | | 2.97 (1.67) |
| Renal and urinary disorders | Proteinuria | | 14 | 9.59 (5.66 - 16.28) | | | 9.55 (105.85) | | 9.44 (6.07) | | 3.24 (2.49) |
|  | Nephrotic syndrome | | 5 | 10.98 (4.54 - 26.59) | | | 10.97 (44.61) | | 10.82 (5.16) | | 3.44 (2.25) |
| Respiratory, thoracic and mediastinal disorders | Dysphonia | | 82 | 31.67 (25.32 - 39.62) | | | 30.86 (2271.93) | | 29.61 (24.55) | | 4.89 (4.56) |
|  | Oropharyngeal pain | | 13 | 3.49 (2.02 - 6.03) | | | 3.48 (22.9) | | 3.47 (2.2) | | 1.79 (1.02) |
|  | Aphonia | | 8 | 16.37 (8.12 - 33.03) | | | 16.33 (112.56) | | 15.99 (8.89) | | 4 (3.02) |
| Skin and subcutaneous tissue disorders | Palmar-plantar erythrodysaesthesia syndrome | | 22 | 14.14 (9.26 - 21.6) | | | 14.05 (261.61) | | 13.8 (9.68) | | 3.79 (3.18) |
|  | Blister | | 17 | 7.55 (4.68 - 12.19) | | | 7.51 (95.07) | | 7.45 (4.99) | | 2.9 (2.21) |
|  | Hyperkeratosis | | 5 | 13.2 (5.44 - 31.99) | | | 13.18 (55.25) | | 12.96 (6.18) | | 3.7 (2.5) |
