# Supplementary material for: A Real‐World Pharmacovigilance Study of Fruquintinib Based on the FDA Adverse Event Reporting System (FAERS) Database
Source: Cancer Med. 2025 Nov 7;14(21):e71352. doi: 10.1002/cam4.71352 (PMC12593544; doi:10.1002/cam4.71352)
Supplement: Supplementary file 5 — Table S3: Signal strength of Fruquintinib‐associated AEs at the PT level in reported cases aged 18–64 years from FAERS data. [file CAM4-14-e71352-s002.docx]

Signal strength of Fruquintinib-associated adverse events at the Preferred Term level in reported cases aged 18–64 years (FAERS database).

| SOC | PTs | Cases | ROR  (95%Cl) | PRR (χ2) | EBGM(EBGM05) | | IC(IC025) |
| --- | --- | --- | --- | --- | --- | --- | --- |
| Blood and lymphatic system disorders | Myelosuppression | 48 | 16.51 (12.37- 22.03) | 16.08 (672.66) | | 15.92 (12.5) | 3.99 (3.57) |
| Endocrine disorders | Hypothyroidism | 6 | 5.72 (2.56- 12.77) | 5.7 (23.19) | | 5.68 (2.9) | 2.51 (1.41) |
| Gastrointestinal disorders | Stomatitis | 21 | 9.52 (6.18- 14.66) | 9.41 (157.13) | | 9.36 (6.52) | 3.23 (2.61) |
|  | Oral pain | 11 | 23.29 (12.81- 42.32) | 23.14 (229.56) | | 22.81 (13.83) | 4.51 (3.67) |
|  | Oral discomfort | 3 | 12.92 (4.14- 40.28) | 12.9 (32.64) | | 12.79 (4.94) | 3.68 (2.22) |
|  | Intra-abdominal fluid collection | 3 | 45.87 (14.53- 144.83) | 45.79 (127.54) | | 44.46 (16.99) | 5.47 (4) |
|  | Tongue discomfort | 3 | 24.7 (7.88- 77.37) | 24.66 (66.99) | | 24.27 (9.34) | 4.6 (3.14) |
|  | Tongue ulceration | 3 | 41.62 (13.2- 131.22) | 41.55 (115.52) | | 40.46 (15.48) | 5.34 (3.87) |
| General disorders and administration site conditions | Death | 147 | 16.59 (14 - 19.66) | 15.25 (1949.15) | | 15.11 (13.11) | 3.92 (3.67) |
|  | Fatigue | 65 | 3.07 (2.4 - 3.94) | 2.99 (87.17) | | 2.99 (2.43) | 1.58 (1.22) |
|  | Asthenia | 33 | 3.76 (2.66- 5.3) | 3.7 (65.28) | | 3.7 (2.77) | 1.89 (1.39) |
| Hepatobiliary disorders | Hepatic function abnormal | 5 | 4.38 (1.82- 10.55) | 4.37 (12.96) | | 4.36 (2.09) | 2.12 (0.94) |
| Infections and infestations | Anal abscess | 4 | 16.12 (6.01- 43.23) | 16.08 (55.99) | | 15.92 (6.98) | 3.99 (2.69) |
| Injury, poisoning and procedural complications | Stoma site haemorrhage | 4 | 93.71 (34.09- 257.61) | 93.49 (344.49) | | 88.05 (37.78) | 6.46 (5.12) |
| Investigations | Blood pressure increased | 44 | 11.2 (8.29- 15.12) | 10.94 (395.22) | | 10.86 (8.45) | 3.44 (3) |
|  | White blood cell count decreased | 14 | 4.35 (2.57- 7.36) | 4.32 (35.69) | | 4.31 (2.77) | 2.11 (1.36) |
|  | Platelet count decreased | 12 | 4.73 (2.68- 8.36) | 4.71 (34.98) | | 4.7 (2.92) | 2.23 (1.43) |
|  | Neutrophil count decreased | 7 | 4.45 (2.12- 9.36) | 4.44 (18.59) | | 4.42 (2.38) | 2.15 (1.12) |
|  | Blood urine present | 4 | 11.31 (4.23- 30.29) | 11.29 (37.24) | | 11.21 (4.92) | 3.49 (2.19) |
|  | Blood albumin decreased | 3 | 19.29 (6.17- 60.31) | 19.26 (51.28) | | 19.03 (7.33) | 4.25 (2.79) |
|  | Blood pressure abnormal | 3 | 6.4 (2.06 - 19.92) | 6.39 (13.59) | | 6.37 (2.46) | 2.67 (1.22) |
|  | Carcinoembryonic antigen increased | 3 | 42.01 (13.32- 132.46) | 41.94 (116.63) | | 40.82 (15.62) | 5.35 (3.88) |
| Metabolism and nutrition disorders | Decreased appetite | 38 | 6.44 (4.67- 8.89) | 6.32 (170.19) | | 6.3 (4.81) | 2.66 (2.19) |
|  | Dehydration | 15 | 6.11 (3.67- 10.17) | 6.06 (63.27) | | 6.04 (3.95) | 2.6 (1.87) |
|  | Hypophagia | 5 | 9.23 (3.83- 22.28) | 9.21 (36.39) | | 9.16 (4.38) | 3.2 (2.01) |
| Nervous system disorders | Neuropathy peripheral | 9 | 3.56 (1.85- 6.86) | 3.55 (16.46) | | 3.54 (2.05) | 1.82 (0.91) |
|  | Taste disorder | 5 | 4.71 (1.96- 11.36) | 4.7 (14.55) | | 4.69 (2.25) | 2.23 (1.05) |
|  | Hypersomnia | 4 | 6.6 (2.47 - 17.65) | 6.59 (18.89) | | 6.57 (2.88) | 2.71 (1.42) |
|  | Cerebral haemorrhage | 3 | 6.91 (2.22- 21.52) | 6.9 (15.08) | | 6.88 (2.66) | 2.78 (1.33) |
| Renal and urinary disorders | Proteinuria | 8 | 14.45 (7.19- 29.03) | 14.38 (98.71) | | 14.26 (7.95) | 3.83 (2.86) |
| Respiratory, thoracic and mediastinal disorders | Dysphonia | 42 | 30.82 (22.62- 41.98) | 30.09 (1158.81) | | 29.52 (22.79) | 4.88 (4.43) |
|  | Aphonia | 6 | 14.45 (6.46- 32.34) | 14.41 (74.16) | | 14.28 (7.28) | 3.84 (2.74) |
| Skin and subcutaneous tissue disorders | Palmar-plantar erythrodysaesthesia syndrome | 18 | 33.01 (20.64- 52.79) | 32.68 (541.09) | | 32 (21.61) | 5 (4.33) |
|  | Blister | 10 | 4.64 (2.49- 8.64) | 4.62 (28.27) | | 4.6 (2.73) | 2.2 (1.33) |
