# Supplementary material for: A Real‐World Pharmacovigilance Study of Fruquintinib Based on the FDA Adverse Event Reporting System (FAERS) Database
Source: Cancer Med. 2025 Nov 7;14(21):e71352. doi: 10.1002/cam4.71352 (PMC12593544; doi:10.1002/cam4.71352)
Supplement: Supplementary file 6 — Table S4: Signal strength of Fruquintinib‐associated AEs at the PT level in reported cases aged ≥ 65 years from FAERS data. [file CAM4-14-e71352-s004.docx]

Signal strength of Fruquintinib-associated adverse events at the Preferred Term level in reported cases aged ≥65 years (FAERS database).

| SOC | PTs | Cases | ROR  (95%Cl) | PRR (χ2) | EBGM(EBGM05) | IC(IC025) |
| --- | --- | --- | --- | --- | --- | --- |
| Blood and lymphatic system disorders | Myelosuppression | 27 | 12.36 (8.43- 18.13) | 12.15 (273.73) | 12.03 (8.73) | 3.59 (3.04) |
| Endocrine disorders | Hypothyroidism | 6 | 5.28 (2.36- 11.79) | 5.26 (20.63) | 5.24 (2.68) | 2.39 (1.29) |
| Gastrointestinal disorders | Haematochezia | 6 | 4.43 (1.99- 9.9) | 4.42 (15.83) | 4.41 (2.25) | 2.14 (1.04) |
|  | Oral pain | 6 | 10.42 (4.66- 23.33) | 10.38 (50.44) | 10.3 (5.25) | 3.36 (2.27) |
|  | Rectal haemorrhage | 6 | 6.02 (2.69- 13.45) | 6 (24.87) | 5.97 (3.05) | 2.58 (1.48) |
|  | Ascites | 4 | 5.62 (2.1 - 15.03) | 5.61 (15.08) | 5.58 (2.45) | 2.48 (1.18) |
| General disorders and administration site conditions | Death | 107 | 3.83 (3.15- 4.67) | 3.62 (206.39) | 3.61 (3.06) | 1.85 (1.56) |
|  | Fatigue | 52 | 2.68 (2.03- 3.53) | 2.62 (52.54) | 2.61 (2.07) | 1.39 (0.98) |
|  | Asthenia | 45 | 4.08 (3.03- 5.5) | 3.99 (101.17) | 3.98 (3.1) | 1.99 (1.56) |
| Hepatobiliary disorders | Jaundice | 3 | 6.51 (2.09- 20.27) | 6.5 (13.88) | 6.47 (2.5) | 2.69 (1.24) |
| Infections and infestations | Kidney infection | 4 | 8.4 (3.14 - 22.48) | 8.37 (25.79) | 8.32 (3.65) | 3.06 (1.76) |
| Injury, poisoning and procedural complications | Underdose | 8 | 8.26 (4.11- 16.6) | 8.22 (50.42) | 8.17 (4.56) | 3.03 (2.06) |
| Investigations | Blood pressure increased | 47 | 11.26 (8.41- 15.08) | 10.92 (420.78) | 10.83 (8.48) | 3.44 (3.01) |
|  | Platelet count decreased | 17 | 4.82 (2.99- 7.79) | 4.78 (50.68) | 4.76 (3.19) | 2.25 (1.57) |
|  | Blood urine present | 5 | 11.6 (4.8 - 28.04) | 11.56 (47.77) | 11.46 (5.47) | 3.52 (2.33) |
|  | Transaminases increased | 3 | 6.87 (2.2 - 21.39) | 6.85 (14.91) | 6.82 (2.63) | 2.77 (1.32) |
|  | Carcinoembryonic antigen increased | 3 | 32.94 (10.44- 103.93) | 32.87 (90.08) | 31.97 (12.22) | 5 (3.53) |
|  | Blood bilirubin increased | 3 | 7.11 (2.28- 22.15) | 7.1 (15.62) | 7.06 (2.73) | 2.82 (1.37) |
| Metabolism and nutrition disorders | Decreased appetite | 28 | 3.68 (2.53- 5.35) | 3.63 (53.37) | 3.62 (2.64) | 1.86 (1.31) |
|  | Hypophagia | 6 | 7.59 (3.4 - 16.98) | 7.57 (33.98) | 7.52 (3.84) | 2.91 (1.81) |
| Neoplasms benign, malignant and unspecified (incl cysts and polyps) | Colon cancer | 21 | 67.55 (43.36- 105.23) | 66.57 (1281.01) | 62.92 (43.42) | 5.98 (5.34) |
|  | Rectal cancer | 9 | 152.55 (75.92- 306.52) | 151.59 (1186.98) | 133.76 (74.6) | 7.06 (6.09) |
|  | Colorectal cancer metastatic | 6 | 128.29 (55.06- 298.91) | 127.75 (677.86) | 114.86 (56.6) | 6.84 (5.69) |
|  | Metastases to lung | 5 | 14.63 (6.04- 35.4) | 14.58 (62.45) | 14.41 (6.88) | 3.85 (2.66) |
| Nervous system disorders | Posterior reversible encephalopathy syndrome | 6 | 28.57 (12.68- 64.33) | 28.45 (155.02) | 27.77 (14.08) | 4.8 (3.69) |
|  | Hypersomnia | 4 | 6.47 (2.42 - 17.32) | 6.46 (18.35) | 6.43 (2.82) | 2.68 (1.39) |
| Renal and urinary disorders | Proteinuria | 10 | 19.45 (10.39- 36.43) | 19.32 (170.89) | 19.02 (11.25) | 4.25 (3.37) |
|  | Nephrotic syndrome | 7 | 39.69 (18.65- 84.48) | 39.5 (253.81) | 38.2 (20.3) | 5.26 (4.21) |
|  | Chromaturia | 3 | 7.2 (2.31 - 22.43) | 7.19 (15.88) | 7.15 (2.76) | 2.84 (1.39) |
| Respiratory, thoracic and mediastinal disorders | Dysphonia | 25 | 13.71 (9.21- 20.41) | 13.49 (285.97) | 13.34 (9.56) | 3.74 (3.16) |
|  | Epistaxis | 8 | 4.52 (2.25- 9.06) | 4.5 (21.69) | 4.48 (2.5) | 2.16 (1.2) |
|  | Aphonia | 5 | 12.07 (4.99- 29.18) | 12.03 (50.05) | 11.91 (5.69) | 3.57 (2.39) |
| Skin and subcutaneous tissue disorders | Palmar-plantar erythrodysaesthesia syndrome | 5 | 7.36 (3.05- 17.76) | 7.34 (27.2) | 7.3 (3.49) | 2.87 (1.68) |
|  | Hyperkeratosis | 4 | 22.74 (8.44- 61.28) | 22.68 (81.29) | 22.26 (9.71) | 4.48 (3.17) |
